# Experimental exploration of a ribozyme neutral network using evolutionary algorithm and deep learning

**DOI:** 10.1038/s41467-022-32538-z

**Published:** 2022-08-17

**Authors:** Rachapun Rotrattanadumrong, Yohei Yokobayashi

**Affiliations:** Nucleic Acid Chemistry and Engineering Unit, Okinawa Institute of Science and Technology Graduate University, Onna, Okinawa 9040495 Japan

**Keywords:** Experimental evolution, RNA, Molecular evolution, Ribozymes

## Abstract

A neutral network connects all genotypes with equivalent phenotypes in a fitness landscape and plays an important role in the mutational robustness and evolvability of biomolecules. In contrast to earlier theoretical works, evidence of large neutral networks has been lacking in recent experimental studies of fitness landscapes. This suggests that evolution could be constrained globally. Here, we demonstrate that a deep learning-guided evolutionary algorithm can efficiently identify neutral genotypes within the sequence space of an RNA ligase ribozyme. Furthermore, we measure the activities of all 2^16^ variants connecting two active ribozymes that differ by 16 mutations and analyze mutational interactions (epistasis) up to the 16th order. We discover an extensive network of neutral paths linking the two genotypes and reveal that these paths might be predicted using only information from lower-order interactions. Our experimental evaluation of over 120,000 ribozyme sequences provides important empirical evidence that neutral networks can increase the accessibility and predictability of the fitness landscape.

## Abstract (web summary)

Neutral networks, which are sets of genotypes connected via single mutations that share the same phenotype, are important for evolvability. Here, the authors provide experimental evidence of a neutral network in an RNA enzyme using a high-throughput assay and deep learning.

## Introduction

The fitness landscape of a biomolecule is a genotype–phenotype map that represents its activity as a function of its sequence space^1^. Molecular evolution can be conceptualized as an adaptive walk along this landscape through a stepwise accumulation of mutations^2^. How the topography of the fitness landscape affects this adaptive walk is an important question in both natural^3^ and artificial evolution^4^. Yet, empirical construction and exploration of these landscapes have proven difficult because of the prohibitively large combinatorial space of biomolecular sequences. However, recent advances in high-throughput sequencing and DNA synthesis have significantly expanded the sequence space amenable to experimental analysis. The fitness landscapes of RNA enzymes, or ribozymes, are particularly important models for molecular evolution, and numerous large-scale empirical mappings of both natural and artificial ribozymes have been reported^5–15^. In addition, because ribozymes play critical roles in the RNA world hypothesis, the topography of their fitness landscapes has important implications regarding the origin of life^16^.

**Fig. 1:**
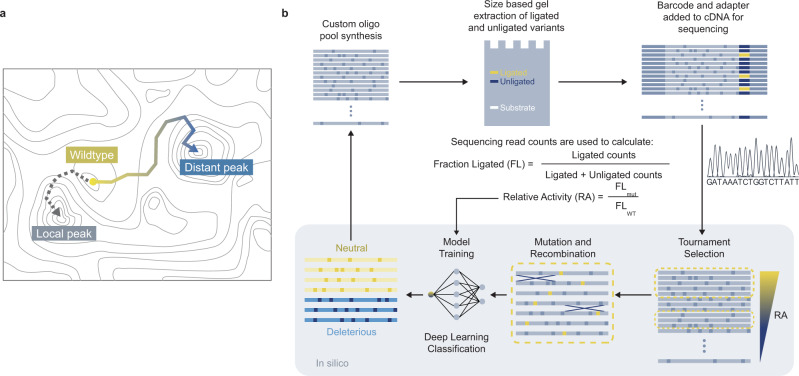
Experimental and computational pipeline for a guided exploration of the F1*U ribozyme fitness landscape. **a** Fitness landscape of a ribozyme is sparsely populated where peaks of activities are separated by extensive valleys of deleterious genotypes. Therefore, evolutionary escape from a local optimum towards a distant peak can be challenging. **b** Outline of ribozyme library assay and evolution process using deep sequencing with in silico selection, recombination, and mutation. Sequences in generation 8 were evolved using a deep-learning classification model.

Many empirical studies on RNA fitness landscapes have revealed that most wild-types (WTs) are located on or near the top of isolated fitness peaks, where only a few mutational steps lead to a significant reduction in fitness^5–8,10,11,13,14^. Two studies that comprehensively mapped almost the entire sequence space of GTP binding^9^ and self-aminoacylating RNAs^12^ further revealed a high degree of ruggedness in RNA fitness landscapes. In these landscapes, fitness peaks were sparsely distributed, and most adaptive walks would be blocked by extensive fitness valleys^9,12^. This indicates that evolution away from local optima and towards distant fitness peaks would be extremely difficult (Fig. 1a)^17^. This empirical evidence contradicts earlier theoretical works that used predicted RNA secondary structures as a proxy for fitness. These computational studies revealed that many connecting sequences can fold into similar structures forming extensive neutral networks^18–20^. A neutral network is a set of genotypes connected by single mutations that share the same phenotype (e.g., structure, catalytic activity). By accessing these networks, evolving molecular populations can travel large mutational distances without detrimental effects on their fitness. Contrasting evidence can also be found in other experimental studies that demonstrated that artificial evolution can be used to engineer a ribozyme that adopts a new structure while retaining its function^21^, or that acquires a new function^22,23^.

The absence of neutral networks in experimental fitness landscapes raises the question of how efficiently the exploration of rugged landscapes can be achieved if most single mutations lead to deleterious mutants. However, most empirical landscapes have been mapped under constant selection and do not represent the dynamic nature of natural evolution. In a natural evolution, changing selection pressure, variable environments, and genetic processes, such as recombination, can influence how fitness landscapes are navigated. These processes have been suggested as efficient ways to cross the fitness valleys^24–26^. Therefore, an important goal is to systematically study how different evolutionary mechanisms can help to efficiently explore rugged fitness landscapes.

In our previous studies, we used on-chip DNA synthesis and high-throughput sequencing to experimentally measure the activities of large libraries of ligase ribozyme variants^15^. The RNA ligase ribozyme under study catalyses the phosphodiester bond formation between the 3ʹ-hydroxyl group of one RNA fragment and the 5ʹ-triphosphate group of another RNA fragment in a template-directed fashion. Ligation chemistry is analogous to that catalyzed by modern RNA polymerase enzymes. Therefore, ligase ribozymes have been extensively studied as models for primitive self-replicating systems. Consequently, ligase ribozymes have much longer artificial evolutionary lineages than any other type of ribozyme^27^. Multiple structural motifs for ligase ribozymes have been discovered, suggesting that RNA sequence space might be well-populated with such phenotypes. This observation implies that neutral networks of ligase ribozymes might be well connected, possibly facilitating access to distant fitness peaks through neutral networks.

In this study, we combine a high-throughput experimental assay and an evolutionary algorithm to explore the empirical neutral network within the catalytic core of a small ligase ribozyme. Starting with the WT, we evolve a population of ligase ribozymes toward distant neutral regions in the fitness landscape through multiple generations. Each generation of ribozyme variants is designed by performing in silico selection, mutation, and recombination of the preceding ribozyme population, whose fitness values were experimentally determined using deep sequencing (Fig. 1b). For the final generation, we create a deep-learning model trained using data from previous generations. We perform 100 rounds of fully in silico selection, mutation, recombination, and fitness estimation based on the model. The population of computationally evolved ribozymes is experimentally evaluated to identify functional ribozyme variants with as many as 17 mutations. We focus on a variant with 16 mutations that exhibits activity comparable to that of the WT. We find that this mutant possessed a robust structural module that could tolerate multiple single and double mutations. Experimental evaluation of all mutational intermediates between the two sequences reveals a remarkable abundance of neutral mutants, with many neutral pathways that could be accessed by single-step mutations. The topography of this region suggests that the prediction of a neutral network could be possible using information from lower-order mutational interactions alone. By experimentally screening over 120,000 ribozyme sequences, we demonstrate that a combination of genetic processes and deep learning can facilitate the exploration of the rugged fitness landscape.

## Results

### Exploration of F1*U ligase ribozyme fitness landscape with an evolutionary algorithm

**Fig. 2:**
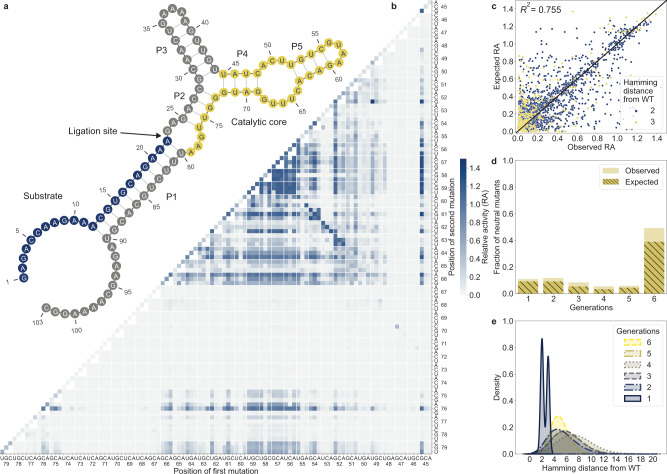
Exploring the fitness landscape of F1*U with high-throughput experimental screening and in silico genetic processes. **a** Predicted secondary structure of the ligated F1*U ligase ribozyme by ViennaRNA 2.5.1 software. The mutated regions consist of the 35 nt catalytic core from U45 to A79 (yellow). The arrow denotes the ligation site. The ligated substrate is depicted in blue. **b** RA of single (diagonal) and double mutants of the F1*U ligase catalytic core. **c** Observed RA from generation 1 compared to the expected RA calculated from the constituent single mutants of each double (blue) and triple (yellow) mutant using the log-additive model. The coefficient of determination (*R*^2^) was calculated from all double and triple mutants and measured how well the model predicted the observed values. **d** Fraction of neutral mutants (RA ≥ 0.2) in each generation of ribozyme sequences. The hatched area indicates the fraction of neutral mutants that were also identified by the epistasis-free log-additive model. **e** Population distribution of the variants in each generation according to their Hamming distances from the WT.

In this study, we explored the fitness landscape of the 35 nt catalytic core of the F1*U ligase ribozyme (Fig. 2a). The F1*U ligase was derived from the catalytic core of the F1 ligase first reported by Robertson and Joyce^28^. Ligated F1*U contains U22A and G80U substitutions introduced in our previous study to allow the analysis of the regiospecificity at the ligation junction^15^. The catalytic core of F1*U contains one terminal loop, one internal loop, P4 and P5 stems, part of the P2 stem, and the GAA bulge that lies close to the ligation junction.

Following our previous study^15^, we used custom on-chip DNA synthesis to generate a template DNA library of the F1*U variants (Fig. 1b). The in vitro transcribed RNA library was placed in a reaction mixture with an excess substrate, which was then separated by denaturing polyacrylamide gel electrophoresis (PAGE). Isolated ligated and unligated ribozymes were extracted from the gel fragments, barcoded by reverse transcription, and processed by PCR to attach sequencing adapters. The library was sequenced using Illumina MiSeq or NovaSeq. Ligated and unligated populations for each variant were identified using the barcodes, and sequencing read counts were used to calculate the fraction ligated (FL) values. Relative activity (RA) was calculated by dividing the FL of each mutant by that of the F1*U WT. Each library was prepared and analyzed twice, and the RA values were calculated using the mean of duplicate measurements (Supplementary Figs. 1, 11a, and 12a). We also calculated the standard deviation of the duplicate RA values (Supplementary Fig. 2). We found almost no correlation between the mean total read count for each variant and the standard deviation (Pearson’s *r* = 0.117) (Supplementary Fig. 2b). For the mean RA and FL, we detected a weak positive correlation with standard deviation (Pearson’s *r* = 0.577 and 0.424, respectively) (Supplementary Fig. 2c, d). For the majority of variants, the standard deviation was smaller than 0.2 (Supplementary Fig. 2a).

Using RA as a proxy for fitness, we designed successive generations of variants using experimental screening combined with in silico selection, recombination, and mutation (see “Methods” for details). For each generation, tournament selection was used, where a variant with the highest RA was selected from a random subset of variants from the previous generation in repeated tournaments until a predetermined number of parents were selected. Genetic diversity was generated by either recombination through one-point crossover, mutation through a single random substitution, or a combination of both. Each new generation was experimentally evaluated using a deep-sequencing assay. Flowcharts detailing the algorithm used to design each generation are provided in Supplementary Figs. 3–5. Each new generation contained only mutants that had not been previously analyzed to maximize the coverage of the sequence space. Eight generations of ribozyme populations were analyzed experimentally. The final generation was designed using a completely in silico evolutionary algorithm guided by a multilayer perceptron model (MLP) trained with the data collected from the preceding generations. The model was trained to classify the variants into neutral (RA ≥ 0.2) or deleterious (RA < 0.2) groups. Using generation 7 as the initial ribozyme population, 100 rounds of in silico evolution were performed. Each round consisted of in silico selection, recombination, and mutation followed by MLP classification. The final population was designated generation 8 (Supplementary Fig. 5). In each generation, we selected individual variants whose RA values were determined using PAGE. The results revealed a good correlation with the RA values determined by sequencing (Supplementary Figs. 6, 11b, and 12b).

### Sequence-activity dataset of the F1*U ligase ribozyme contained epistatic and structural information

We experimentally screened a library of all 105 single, all 5355 double, and 4540 randomly chosen triple mutants of the WT F1*U to serve as a starting point. The double mutant map indicated that almost all substitutions were tolerated in the terminal loop between G56 and A59 (Fig. 2b). The P5 stem was also relatively tolerant to mutations, especially compensatory substitutions that maintained base pairing. Mutation 45 G appeared to have a strong positive effect on other neutral mutations. Mutation 76 C was also well-tolerated, possibly because it stabilized the P2 stem by base pairing with 25 G. Mutations in the GAA bulge were surprisingly well-tolerated considering its proximity to the ligation junction. However, mutations within the P4 and P2 stems resulted in a complete loss of activity.

Epistasis is observed when mutational effects are combined in a nonlinear manner. Epistasis is an important indicator of landscape ruggedness and the accessibility of evolutionary paths^29–32^. Therefore, we analyzed epistasis around the WT by calculating the expected RA for each mutant from its constituent single mutants using the log-additive model. In this model, the expected ln(RA) of a mutant was calculated as the sum of ln(RA) of all its constituent single mutants. A perfect correlation between the observed and expected RA values in this model indicates the absence of epistasis. The fitness landscape within the first three mutations of F1*U was relatively smooth, with 75.5% of the variants, particularly the double mutants, being predictable without epistasis (Fig. 2c). The low level of epistasis suggests that the evolutionary path away from the WT may not be as severely constrained as observed in earlier studies of fitness landscapes^29–31^.

### In silico genetic processes, particularly recombination, increase the probability of finding neutral mutants

Next, we investigated whether a combination of in silico selection, recombination, mutation, and in vitro experimental screening could be used to identify neutral mutants further away from the WT in the fitness landscape. Previous empirical studies have shown that functional genotypes are extremely rare in RNA fitness landscapes^7,9–12^. Similarly, most genotypes in the F1*U fitness landscape were nonfunctional, with most variants in generations 1–5 having an RA well below 0.04 (Supplementary Fig. 7). For this study, we set an RA of 0.2 as the threshold for the neutral phenotype based on the following reasoning. First, the standard deviation of the RA values estimated by deep sequencing for the vast majority of the variants was less than 0.2 (Supplementary Fig. 2a). We also found RA ≥ 0.2 could be reliably detected by the PAGE assay. It could be argued that the threshold of RA ≥ 0.2 is too low to be considered “neutral”. However, fitness is highly context-dependent. Even though a small difference in catalytic activity could result in rapid extinction of the weaker genotype when selection occurs at the molecular level (i.e., not neutral), any activity above a certain threshold would be selectively neutral if the catalyst is part of a protocell whose replication rate is limited by another step (e.g., reproduction of the compartment).

Generations 2–6 were successively designed using combinations of in silico selection, recombination, and mutation. The experimental analysis of generations 2 and 3 revealed a very low fraction of neutral mutants (Fig. 2d). We increased the recombination frequency and population size in generations 4 and 5, hoping to increase the chance of detecting more neutral mutants in distant regions of the landscape (Fig. 2e). However, this resulted in even lower fractions of neutral mutants. The algorithm used to design generations 2–5 performed the recombination of two selected parents, with the resulting recombinant immediately undergoing a random point mutation (Supplementary Fig. 3). Random mutations are more likely to lead to deleterious mutants than purely recombining parental genotypes that are already known to be neutral. Owing to the algorithm design, most of the variants experimentally assayed in generations 2–5 also underwent random substitutions. This could explain why generations 2–5 had a very low fraction of neutral mutants (Fig. 2d).

To address this issue, we modified the algorithm for generation 6 onward. Following tournament selection, a set of recombinants was generated without substitutions. Another set of variants was then created by selecting random variants from a pool of parents and recombinants to undergo random substitution. The final population comprised a combination of pure recombinants and random mutants (Supplementary Fig. 4). The major difference in this new strategy was that most variants in generation 6 onward were generated from the recombination of parental sequences alone, with only a small fraction generated by a random substitution applied to a parent or recombinant. The exact number of pure recombinants and random mutants created in each generation are listed in Supplementary Table 1. This new algorithm dramatically increased the fraction of neutral mutants to almost 0.5 in generation 6. This supports earlier observations in directed evolution experiments in which recombination was shown to better preserve function and structure than random substitution^24,33^. Recombination of selected neutral mutants is more likely to result in a neutral mutant if fitness is a result of linear combinations of mutational effects (i.e., when there is no epistasis). Indeed, most of the neutral mutants identified in each generation were also identified from the expected RA values using the log-additive model (Fig. 2d, hatched bars). This indicates that the current algorithm identified neutral mutants that were mostly free of epistasis.

### Machine-learning-guided evolutionary algorithms could discover neutral mutants in distant and epistatic regions of the fitness landscape

**Fig. 3:**
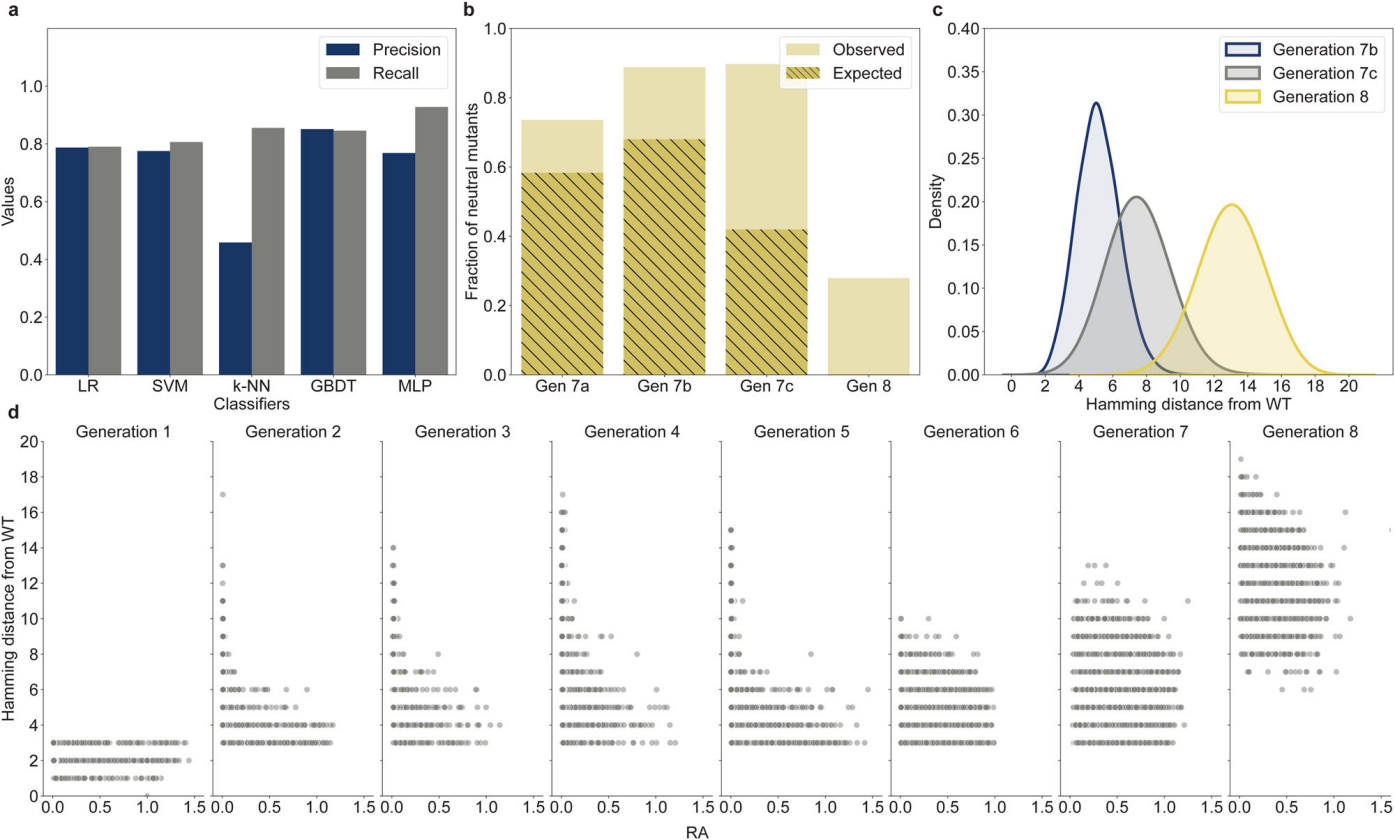
Machine-learning-assisted evolutionary algorithm enables in silico evolution towards distant regions of the fitness landscape. **a** Comparison of classification metrics for logistic regressions (LR), k-nearest neighbors (k-NN), support vector machine (SVM), gradient-boosted decision trees (GBDT), and multilayer perceptron (MLP). The models were trained on 20,920 variants from generations 1–6. Precision and recall were evaluated from a model prediction on a held-out testing set of 8967 variants. **b** Fraction of neutral mutants identified by different strategies. Generation 7a used in silico selection, recombination, and mutation only. Generation 7b was generated by the in silico genetic process, and only those that were predicted to be neutral by MLP were selected. Generation 7c was created by shuffling generation 7b with a 10× recombination rate. and only variants predicted to be neutral by the MLP were selected. Generation 8 was generated using a completely in silico evolutionary algorithm involving 100 rounds of selection, recombination, mutation, and MLP classification. The hatched area indicates the fraction of neutral mutants that was also identified by the epistasis-free log-additive model. **c** Population distribution of generation 8 compared to generations 7b and 7c according to Hamming distance from the WT. **d** RA and Hamming distances of all 53,823 mutants screened over eight generations.

Next, we tested whether machine-learning models could learn epistatic information from the dataset collected thus far and predict neutral mutants in distant regions. Using data from generations 1–6, we trained a group of models to classify the variants as neutral or deleterious. Because it was difficult to predict which model would best fit the data, we decided to test several popular models with varying degrees of complexity. Logistic regression (LR) and support vector machine (SVM) with linear kernels are linear models used as performance baselines. The k-nearest neighbor (k-NN) and gradient-boosted decision trees (GBDT) are powerful nonlinear models that can learn complex interactions, such as epistasis in the data. Finally, an MLP is a neural network model that can potentially learn complex nonlinearities, such as higher-order epistasis. A more detailed description and comparison of each model is in “Methods”. To select the model to be incorporated into the algorithm, precision and recall were used as performance metrics. Precision is the fraction of positive (neutral) predictions that are true positive. This represents the probability that the variants predicted to be neutral are actually neutral when tested experimentally. Recall is the fraction of neutral mutants in the experimental data identified by the model. All models performed relatively well, with MLP performing the best in terms of recall (0.93) while maintaining good precision (0.77) (Fig. 3a). MLP recall also outperformed other models in the prediction of neutral mutants with higher Hamming distances from the WT (Supplementary Fig. 8). Classification models often have a trade-off between recall and precision. We focused on recall as the key metric, while allowing for a small trade-off in precision to maximize our chance of identifying rare neutral mutants.

We then designed three populations to test whether MLP could be used to increase the fraction of neutral mutants. For generation 7a, because we were confident that pure recombinants were more likely to be neutral than random mutants, we increased the proportion of pure recombinants in the population to 80% from ~66% in generation 6 (Supplementary Table 1). Another smaller set of variants was created in the same manner as in generation 7a, except that only the offspring predicted to be neutral by the MLP were selected for the final population (generation 7b). To determine whether the MLP prediction remained accurate at higher Hamming distances, another test set was created by recombining generation 7b at an average of 10 recombination events per variant. The variants created from this procedure were only selected if predicted to be neutral by the MLP (generation 7c). The fraction of neutral mutants increased to 0.74 in generation 7a, possibly because of the larger pool of neutral parents from generation 6 and increased proportion of pure recombinants (Fig. 3b). MLP classification increased the fraction of neutral mutants to 0.89 in generation 7b. The fraction of neutral mutants remained the same, even with a higher rate of recombination in generation 7c (Fig. 3b, c). This indicated that despite sampling further away from the WT, the combination of in silico selection, recombination, mutation, and MLP could identify neutral mutants with high accuracy (Fig. 3b, c).

Next, we tested whether the information from our experimental screening could be used to identify neutral mutants with larger Hamming distances from the WT. We created a complete in silico evolutionary algorithm using the MLP model, which was retrained with data collected from all seven generations. Starting from generation 7, we performed 100 rounds of in silico selection, recombination, mutation, and MLP classification to create generation 8, in which all variants were predicted to be neutral (Supplementary Fig. 6). Experimental analysis showed that the fraction of neutral mutants in generation 8 was only 0.28 (Fig. 3b). However, the average Hamming distance from the WT in generation 8 was 13, which was significantly higher than that of the preceding generations (Fig. 3c). The drop in MLP accuracy was expected, because the model made predictions for variants with higher Hamming distances than those observed during training. However, the fraction of neutral mutants was still significantly higher than that in generations 1–5 despite their lower average Hamming distances (Fig. 2d, e). We identified neutral mutants with as many as 17 mutations and numerous mutants with RA comparable to the WT across 16 mutational steps (Fig. 3d).

In generation 7c, less than half of the neutral mutants could be identified using the log-additive model (Fig. 3b, hatched bars). This suggests an increasing contribution from epistasis at higher Hamming distances. Epistasis limited the efficiency of our algorithm because the recombination and mutation of selected neutral mutants were less likely to lead to more neutral mutants because of the nonlinear combination of mutational effects. In generation 8, almost all neutral mutants identified were expected to be deleterious under the epistasis-free model. This indicated that the neural network was able to learn higher-order, nonlinear mutational effects, and identify distant neutral mutants that would have been inaccessible through in silico selection, mutation, and recombination alone.

### Evolution along the F1*U neutral network led to a confined region of higher mutational robustness

**Fig. 4:**
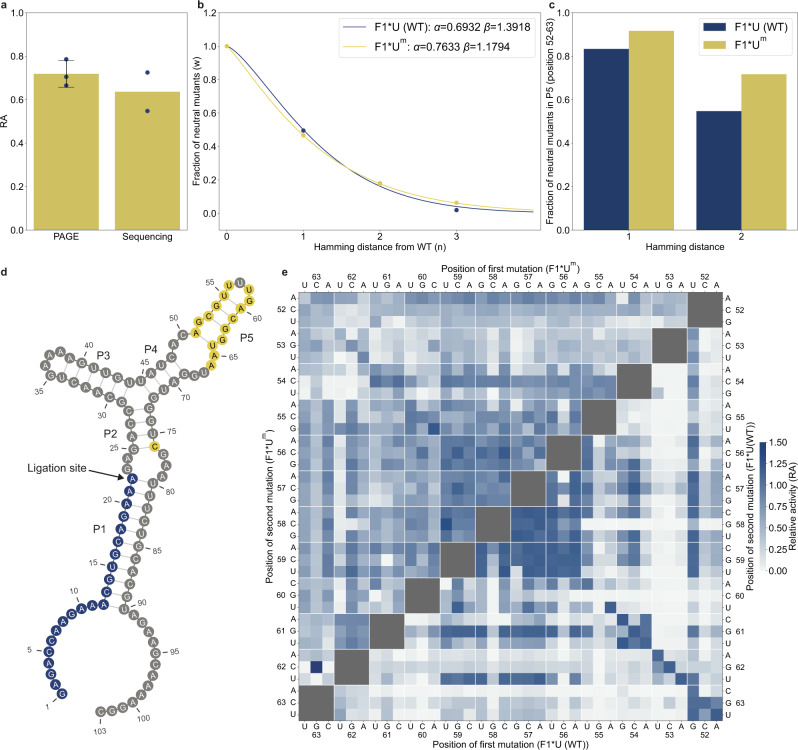
F1*U^m^ has a more mutationally robust P5 stem than F1*U. **a** Comparison between RA values of F1*U^m^ determined by PAGE and sequencing. PAGE experiments were performed in triplicate, and sequencing experiments were performed in duplicate. Data are presented as mean value for sequencing experiments and mean value +/− SD for PAGE experiments. **b** The decline in the fraction of neutral mutants ($\omega$) due to mutation accumulation ($n$) fitted to the directional epistasis model $\omega \left(n\right)={e}^{-\alpha {n}^{\beta }}$ by the nonlinear least square method (SciPy, Python). $\alpha$ is the decay parameter where lower α indicates higher mutational robustness. $\beta$ is strength of directional epitasis. $\beta$ > 1 indicates an excess of negative epistasis and $\beta$ < 1 indicates an excess of positive epistasis. $\beta$ equal to 1 indicates a balanced mix of positive and negative epistasis or there is no epistasis. **c** Fraction of neutral single and double mutants in the P5 stem of F1*U (blue) and F1*U^m^ (yellow). **d** Predicted secondary structure of the ligated F1*U^m^ by ViennaRNA 2.5.1 software. The 16 mutated positions are yellow, and the substrate is blue. **e** RA of double mutants in the P5 stem of F1*U (lower triangle) and F1*U^m^ (upper triangle).

Computational and experimental evidence suggests that the accumulation of neutral mutations can lead to increased mutational robustness^20,34,35^. Therefore, we sought to determine whether a mutant evolved by our algorithm gained mutational robustness. We selected a mutant, F1*U^m^, which had the highest Hamming distance (16) while retaining catalytic activity comparable to the WT (RA = 0.63). The RA of F1*U^m^ was 0.72 when individually assayed using PAGE (Fig. 4a). F1*U^m^ was also predicted to fold into a secondary structure that was very similar to that of the WT (Fig. 4d). The mutations in F1*U^m^ were almost exclusively within the P5 stem loop, with an additional 76 C mutation. The mutational tolerance of the P5 stem loop was consistent with previous reports showing that ligase activity is retained even when the P5 stem is removed or replaced with aptamer sequences^15,36^.

We experimentally assayed all 105 single, all 5355 double, and 4540 random triple mutants of F1*U^m^. Then we fitted the data to the equation $\omega (n)={e}^{-\alpha {n}^{\beta }}$, which is a directional epistasis model^37^. The model fits the fraction of neutral mutants $\omega$ at the Hamming distance *n* from the reference sequence to mutational robustness *α* and directional epistasis *β* (“Methods”). Fitting the single-, double- and triple-mutant data for both F1*U (generation 1) and F1*U^m^ showed that *α* and *β* were highly similar for both genotypes (Fig. 4b). This indicated that the two genotypes had similar overall mutational robustness. Directional epistasis (*β*) was greater than 1 in both cases, indicating an excess of negative epistasis consistent with the landscapes of previously studied ribozymes^38^.

However, we found that the fractions of neutral single and double mutants in the P5 region were higher in F1*U^m^ than in the WT (Fig. 4c). A comparison of the double mutant maps within the P5 stem loop also revealed that some non-compensatory mutations that were deleterious in the WT were neutral in F1*U^m^ (Fig. 4e). These results indicated that the effects of neutral mutants could be highly confined. The stabilizing effects of mutations in one region may not be sufficient to reduce sensitivity to mutations in other parts of the molecule.

### Combinatorial space between F1*U and F1*U^m^ contains an extensive neutral network

**Fig. 5:**
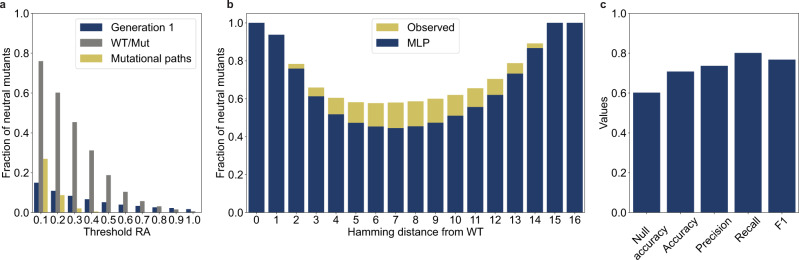
Combinatorial space between F1*U and F1*U^m^ contains many neutral mutants predictable by deep learning. **a** Fraction of neutral mutants in the combinatorial library between the F1*U and F1*U^m^ (WT/Mut) and in generation 1 according to different RA thresholds of neutrality. A total of 10^6^ unique mutational paths were randomly sampled that could transform the WT sequence to F1*U^m^ in 16 direct mutational steps. Paths were considered neutral if none of the steps resulted in a mutant with RA lower than the neutrality threshold. **b** Fraction of neutral mutants at each Hamming distance in the WT/Mut library as measured by experimental assay (yellow). The blue area indicates the fraction of the neutral mutants that was also identified by MLP trained on data from generations 1–7. **c** Performance metrics of MLP when used to classify the WT/Mut library.

The structural and functional neutrality between F1*U (WT) and F1*U^m^ suggests that these two variants are connected by many accessible mutational pathways. To confirm this, we synthesized the entire combinatorial space within the 16 mutated positions of F1*U^m^ to obtain a library of 65,536 (2^16^) variants (WT/Mut). Experimental screening of the library showed that the fraction of neutral mutants in this region was significantly higher than that of the single, double, and triple mutants of the WT screened in generation 1 (Fig. 5a). At the neutrality threshold of 0.2, the fraction of neutral mutants was 0.11 in generation 1 compared to 0.60 in the WT/Mut library. Many mutants with an RA similar to that of the WT were also identified across different Hamming distances (Supplementary Fig. 13). Furthermore, we identified many accessible single-step mutational paths with nearly 10% of 10^6^ randomly sampled paths accessible at a neutrality threshold of 0.2 (Fig. 5a). We also identified 39 paths that maintained RA above 0.6. These results revealed that F1*U and F1*U^m^ are indeed connected by neutral networks, and many accessible paths exist that connect the two genotypes through sequential substitutions.

Next, we assessed the predictability of this landscape using the same MLP model used to design generation 8, with no additional training, to classify the variants in the neutral network. Despite having seen only 441 mutants (~0.7%) in this neutral network, the model was able to identify most of the neutral mutants across all Hamming distances (Fig. 5b). The accuracy of the model was 0.71, substantially higher than the accuracy of generation 8 (0.28 accuracy) (Fig. 5c). This suggests that the model was overfitted to this neutral network. However, the accuracy was higher than the null accuracy (0.60) with balanced recall and precision (F1 score = 0.77). A null accuracy is achieved if the model predicts the majority class for all variants. Because the model outperforms null accuracy, we can assume that it is not simply biased towards making a positive prediction for the neutral network. The improved accuracy of the MLP could be attributed to the reduced diversity of the WT/Mut library compared to generation 8 (average Hamming distance = 8 vs. 13). However, the manner in which mutations interact also determines landscape predictability. Therefore, we investigated how nonlinear interactions of mutations or epistasis could influence the predictability of the neutral network between F1*U and F1*U^m^.

### The neutral network between F1*U and F1*U^m^ is relatively smooth compared to the region sampled by the evolutionary algorithm

**Fig. 6:**
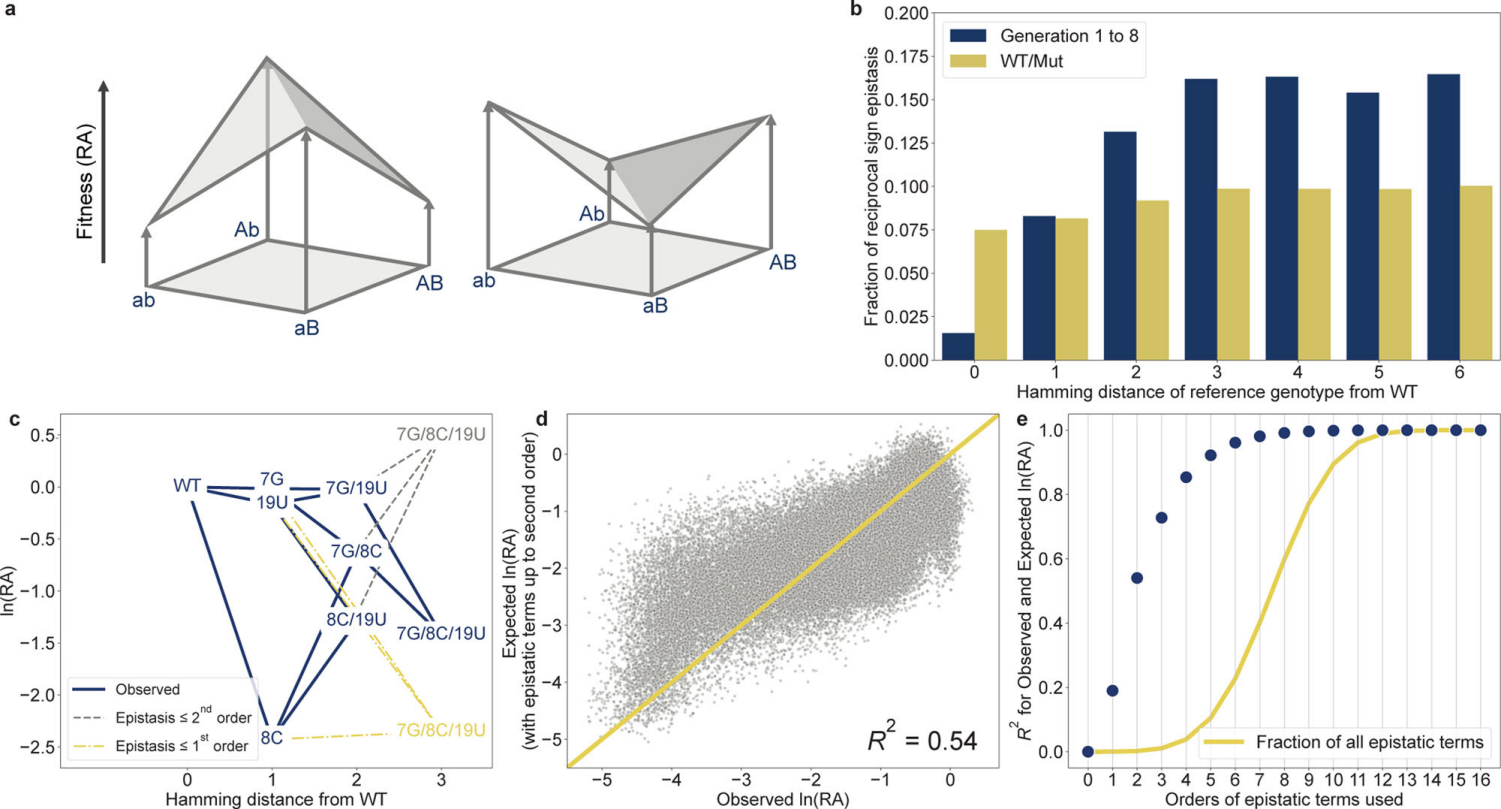
Neutral network between F1*U and F1*U^m^ is relatively smooth at higher Hamming distance with fitness largely influenced by lower-order mutational interaction (epistasis). **a** Illustration of reciprocal sign epistasis involving two genotypes that differ by two substitutions (ab and AB). Reciprocal sign epistasis is observed when both the reference genotype (ab) and the double mutant (AB) has higher or lower fitness than both intermediate single mutants (Ab and aB). This leads to landscape ruggedness and can restrict evolutionary paths. **b** Fraction of pairwise reciprocal sign epistasis in generation 1 to 8 (blue) and WT/Mut library (yellow) categorized by the Hamming distance of the reference genotype ab from the WT. Higher fractions indicate the higher ruggedness of the landscape. **c** Diagram illustrating ln(RA) of the sub-landscape between the WT and a triple-mutant 7G/8C/19U. Experimentally observed values are in blue. ln(RA) of the triple mutant when only first-order WT-relative epistatic terms were considered is in yellow. ln(RA) of the triple mutant when only first- and second-order WT-relative epistatic terms were considered is shown in gray. **d** Observed ln(RA) values of WT/Mut library and the expected ln(RA) from a background-averaged epistatic model that only includes the first- and second-order epistatic terms. **e** Coefficients of determination (*R*^2^) values between the observed ln(RA) and the expected ln(RA). Expected ln(RA) was calculated at each step by cumulatively adding background-averaged epistatic terms of successively higher order. For each step, *R*^2^ scores were calculated using all the variants in the library. The fraction of all epistatic terms used to calculate the expected ln(RA) at each step is shown by the yellow line.

Smooth neutral paths can potentially help evolution traverse an otherwise rugged fitness landscape. To investigate this, we quantified the ruggedness of the neutral network between F1*U and F1*U^m^, and compared it to that of the nearby sequence space. Pairwise reciprocal sign epistasis occurs between two genotypes that differ by two mutations when both genotypes exhibit lower or higher fitness than their two intermediate single mutants (Fig. 6a). Reciprocal sign epistasis is a particularly severe form of epistasis that can restrict evolutionary paths within the fitness landscape^29,31^. Therefore, a high proportion of reciprocal sign epistasis has been used as an indicator of landscape ruggedness^39,40^.

First, we searched for all unique 2^2^ subgraphs representing pairs of sequences that differed by two mutations and their intermediate single mutants (Fig. 6a). In total, we identified 214,068 subgraphs in generations 1–8 and all 3,932,160 possible subgraphs in the WT/Mut library. Next, we designated the sequence in each subgraph with the smallest Hamming distance to the WT as the reference genotype “ab”. We then determined whether each subgraph exhibited reciprocal sign epistasis. The analysis showed that the fraction of reciprocal sign epistasis increased substantially at higher Hamming distances in the sequences sampled between generations 1 and 8 (Fig. 6b). Between Hamming distances 1–6, the fraction of reciprocal sign epistasis in generations 1–8 ranged from ~0.08 to ~0.175 compared to less than 0.1 in the WT/Mut dataset. We also identified reciprocal sign epistasis at Hamming distances greater than 6. However, the total number of subgraphs identified at these distances was too low for a reliable comparison (Supplementary Table 2). The dataset from generations 1–8 represents a biased and partial sampling of the fitness landscape, and only indicates the ruggedness of the sequence space sampled by the evolutionary algorithm. However, our results indicate that the neutral network between F1*U and F1*U^m^ appears to be quantitatively smoother than the nearby sequence space, which may facilitate evolution.

### Epistasis within the neutral network between F1*U and F1*U^m^ is largely captured by lower-order interactions

Higher-order epistasis has been shown to substantially influence the topography and predictability of fitness landscapes^41^. Therefore, we chose to explore the effects of higher-order epistasis in the neutral network between F1*U and F1*U^m^. Epistasis of any order can be investigated by examining how a combination of different numbers of mutations gives rise to the RA of each variant. We examined the variant 7G/8C/19U as an example (Fig. 6c). Mutational effects can be observed by mapping the RA values to log space, where a variant with higher or lower activity than the WT results in ln(RA) > 0 or ln(RA) < 0, respectively. The first-order interaction or epistatic terms are simply ln(RA) for the single mutants 7G, 8C, and 19U. If all mutations act independently, then ln(RA) of the triple mutant will be the sum of the first-order terms (yellow). This is the same as the log-additive model used in previous sections. Nonlinearity arises if mutational effects are combined non-independently. If pairs of mutations interact, then ln(RA) of the triple mutant depends on second-order terms (pairwise epistasis). This can be quantified as the difference between the ln(RA) of a double mutant and the sum of ln(RA) of its constituent single mutants. Adding up all the second- and first-order terms provide ln(RA) of the triple mutant if only pairwise interactions are present (gray). However, the observed ln(RA) of 7G/8C/19U was different from both scenarios (blue). This indicated that there was a contribution from third-order epistasis, and the combined effects could not be estimated only from the second- or first-order terms. This is the influence of higher-order epistasis, which limits fitness landscape predictability.

With a combinatorially complete neutral network, we can investigate epistasis by extending the calculation described above across all interaction orders. In the above example, epistasis was calculated when mutational effects were considered relative to a single reference, the WT. We can also measure epistasis when mutational effects are averaged across all genotypic backgrounds that occur using the Walsh–Hadamard (WH) transform (see “Methods” for details)^42^. This method has been shown to capture the interactions across the entire sequence space better than the single-reference method^7,41,43^. Importantly, this operation is a linear transformation of the RA values into non-additive epistatic terms. Therefore, the RA values can be retrieved by performing an inverse operation on the epistatic terms.

We calculated the WH transform of the WT/Mut library and then reconstructed the ln(RA) values with all epistatic terms higher than the second-order set to zero. The *R*^2^ (coefficient of determination) score of the reconstructed and observed ln(RA) was 0.54 (Fig. 6d). This indicates that the background-averaged first- and second-order epistatic terms, which comprise only 0.2% of all epistatic terms, could explain the fitness effects of more than half of the landscape. To achieve an almost perfect prediction, background-averaged epistatic terms up to the 7th order were required (Fig. 6e). When the *R*^2^ values were calculated using only variants with Hamming distance higher than the maximum order of the included interaction terms, the results remained qualitatively unchanged (Supplementary Table 3). When epistatic terms were calculated from mutational effects that were only considered in the background of the WT, as depicted in Fig. 6c, almost all epistatic terms were required to achieve similar accuracy (Supplementary Fig. 14). Consistent with other works^7,43,44^, epistatic information was encoded in much fewer terms when mutational effects were averaged across different genotypic backgrounds. Although analysis of background-averaged epistasis up to the 7th order would be inaccessible for most experimental set-ups, a significant gain in predictive power can be achieved by including only the 3rd or 4th order background-averaged epistatic terms. In fact, only the 3rd order terms were required to achieve an accuracy similar to that of the current MLP model (*R*^2^ ≈ 0.72). These results suggest that for this neutral network, MLP can achieve high prediction accuracy by learning the background-averaged terms of lower-order interactions. Successive rounds of selection, recombination, and mutation could potentially facilitate this process without the knowledge of the complete landscape. By retaining mutational combinations that were persistently neutral after rounds of diversification, MLP could learn which combinations of mutational effects remained significant in different genetic backgrounds.

Finally, some of the nonlinearities identified in this library could be attributed to global noise from experimental errors. We used the RA values calculated from the mean of the two replicates to reduce experimental errors. Furthermore, any global nonlinearities would also have affected the MLP predictions, because the same dataset was used to train it. Therefore, our conclusion remains robust in terms of the predictability of this dataset using the current model. Further statistical tests could be performed to remove global nonlinearities, which could improve model performance^43,45,46^.

## Discussion

The fitness landscapes of ribozymes can provide important information about the emergence of catalytic properties and how they could evolve toward more complex living systems^16,18,47,48^. Of particular interest is how catalytic genotypes are distributed within the landscape, and recent observations suggest that these are rare and sparsely distributed^5,12^. Therefore, accessing these genotypes would be challenging even when starting from a genotype with a known function. Here, we implemented a simple evolutionary algorithm that, at its optimum, could identify functional genotypes with almost 90% efficiency. Accurate prediction of fitness landscapes by machine-learning models are limited by epistatic effects. Collecting informed training data is already difficult because of “holey” fitness landscapes^49^. The rarity of functional genotypes indicates that random sampling of the ribozyme fitness landscape would yield a dataset that is highly biased towards deleterious (nonfunctional) variants. The scarcity of positively labeled data limits the amount of learning that can be achieved using machine-learning models. We overcame these problems by using in silico selection, recombination, and mutation to guide adaptive walk along paths that are smooth and relatively free of epistasis. This generated a dataset with a more balanced distribution of neutral and deleterious mutants. This dataset provided information on key combinations of mutations that are neutral in different mutational backgrounds. This information can then be learned by a deep neural network to identify functional variants in distant regions using data acquired only from the first few mutational steps.

In this study, we tested several popular machine-learning models with no optimization beyond the default hyperparameters provided by the software package. MLP outperformed other models by a small margin (Fig. 3a) and was used for subsequent analysis. However, it should be noted that the other tested models also performed relatively well, and further optimization of the hyperparameters may yield improved performance possibly with less overfitting. Large-scale experimental fitness landscape data such as those generated in this study have become available only relatively recently. More focused studies that systematically compare how well various models—with optimized hyperparameters—can capture the characteristics of the experimental fitness landscape data are needed to elucidate which types of models are better suited for analyzing such datasets.

The relative ease with which our algorithm traversed this landscape contradicts previous observations from other landscapes in which long evolutionary paths are mostly blocked by deleterious mutants^6,7,9–13,17,30,50^. We suspect that large neutral networks within our landscape could facilitate adaptive walk. To investigate this, we mapped all 65,536 mutational intermediates between two neutral mutants separated by 16 mutations, F1*U and F1*U^m^. We demonstrated that the two sequences are connected by an extensive neutral network. The combinatorial space is highly abundant in functional mutants and contains many accessible evolutionary paths.

We discovered an extensive neutral network between the structurally and functionally similar F1*U and F1*U^m^. This suggests that neutral networks might be more common among ribozymes of the same family, but this may not always be the case. The most comprehensive ribozyme fitness landscapes published thus far is of a 21 nt self-aminoacylating ribozyme^12^. In this study, very few viable pathways were found even among closely related motifs. The best pathway involved a variant with almost a tenfold reduction in activity. This suggests that even amongst ribozymes of the same family, large extensive neutral networks, such as those found in this study, can be surprisingly rare. The neutral network between ribozymes which are more structurally and functionally different could be even rarer. Another study resulted in the construction of the complete combinatorial landscape within 14 mutated positions of an 88 nt ribozyme that could transform a ligase structural motif into a self-cleaving motif^6^. Mutational paths within this landscape were highly constrained, and activity diminished significantly after only a few mutational steps away from either motif.

It is important to understand how evolutionary adaptation and innovation can occur if neutral networks are rare. In this study, we showed that the adaptive paths between F1*U and F1*U^m^ led to a more mutationally robust P5 stem. Our combinatorial map was limited to 16 mostly contiguous positions within the ribozyme. The robustness and high connectivity of this confined region implies that it could be a good starting point for the evolution of a new function or adaptation to a new environment. This conclusion is supported by another study which showed that a self-splicing ribozyme possesses an intramolecular buffer module that can accumulate large number of mutations. These mutations alter the phenotype of the ribozyme upon exposure to a stressful environment, possibly providing a way for evolutionary adaptation^8^. Altogether, our data support the theory that evolutionary innovation and adaptation are more likely to happen through the expansion of small contiguous motifs rather than through sudden large-scale structural changes^51^.

Finally, we focused on the mutational interactions that govern the topography and predictability of this neutral network. Earlier studies have shown that fitness landscapes can be encoded into sparse background-averaged interaction terms and can be determined from a small fraction of key mutational interactions^43,52^. Similarly, our results showed that the topography of this neutral network is largely encoded within lower-order background-averaged interaction terms. Other studies have leveraged this sparsity alongside knowledge from the field of compressed sensing (CS) to better predict fitness values from small sample sizes^43,44,52^. Our algorithm, which utilized genetic processes, was also able to predict distant genotypes despite being trained mostly on lower-order mutants. This offers a potentially simpler approach that could also be relevant for understanding how early evolution identified key interaction terms during its navigation of the fitness landscape.

The astronomical size of the fitness landscape implies that we will probably never be able to map it in its entirety. However, the observations we made here, including that the prediction of the fitness landscape across large distances is possible, especially within neutral networks, offer hope that extrapolation from small sampling is possible. Finally, the large sequence-activity dataset that we generated, particularly the empirical evidence of a neutral network, warrants further quantitative analysis. In particular, whether the properties we have discovered are unique to this neutral network and whether information learned here can be used to predict other parts of the landscape should be determined. This may have important implications in areas ranging from molecular engineering^53^ to viral evolution^54^.

## Methods

### Preparation of ligase ribozyme libraries

Commercially available custom oligo pools (Twist Biosciences) were used to construct dsDNA templates of ribozyme libraries for in vitro transcription. The oligo pools were ordered with the T7 promoter and ribozyme sequence, which were amplified by PCR using primers Ligase-lib-f and Ligase-lib-r (Supplementary Data 1) and Phusion High-Fidelity PCR Master Mix with HF Buffer (New England Biolabs (NEB)). The PCR product was column-purified using the DNA Clean & Concentrator-5 kit (Zymo Research). In vitro transcription was performed using a purified dsDNA template with a ScriptMAX Thermo T7 Transcription Kit (Toyobo) in a volume of 10 µL. After the transcription reaction, the solution was incubated for 10 min at 37 °C with a DNase-I (NEB) solution consisting of 2 µL DNase I (2 U/μL), 2 µL 10× DNase I Reaction Buffer, and 6 µL nuclease-free water. The RNA product was column-purified using the RNA Clean & Concentrator-5 kit (Zymo Research).

### Ligation reactions of ribozyme libraries

The ribozyme pool (0.8 μM) was mixed with substrate F1*subA (Supplementary Data 1) at 8 μM in nuclease-free water in a reaction volume of 24 µL. The solution was heated to 72 °C for 3 min, and then cooled to 4 °C for 5 min. The RNA solution and the 4× reaction buffer (200 mM EPPS pH 7.5, 2.0 mM MgCl_2_, 8 U/μL RNase Inhibitor, Murine (NEB)) were separately incubated at 37 °C for 3 min. The reaction was initiated by adding the RNA solution to 8 µL of reaction buffer, followed by incubation for 60 min at 37 °C. The reaction was terminated by adding 72 µL of cold stop solution (25 µL 0.5 M EDTA and 65 µL RNA Loading Dye (2×) (NEB)) and kept on ice.

### Preparation of sequencing templates

The reaction solutions were heated to 95 °C for 3 min and separated on a 12% urea polyacrylamide gel. The gels were stained with SYBR Gold (Thermo Fisher) and visualized using a blue light transilluminator. Ligated and unligated ribozymes bands were excised and crushed. RNA was extracted in Tris/NaCl buffer (30 mM Tris-HCl, pH 7.5, 30 mM NaCl) by shaking at 1200 rpm and 4 °C for 18 h. RNAs were precipitated by ethanol using Quick-Precip Plus Solution (EdgeBio), washed twice with 70% ethanol, and resuspended in nuclease-free water. The ligated and unligated RNA was dissolved in 10 µL of nuclease-free water, and 5 μL was used for reverse-transcription reactions. Reverse-transcription reactions were performed in a 10 µL volume with Maxima H Minus Reverse Transcriptase (Thermo Fisher) according to the manufacturer’s instructions. R1-[barcode]-F1-lig (Supplementary Data 1) was used as the reverse-transcription primer. Different barcodes were used for unligated and ligated ribozymes. Reverse-transcription reactions were allowed to proceed for 30 min at 65 °C, and the enzyme was inactivated at 85 °C for 5 min. To remove the primers, 1 µL of 20 U/µL exonuclease I (NEB) was added to the reverse-transcription solution and incubated for 30 min at 37 °C followed by 15 min at 85 °C. The solutions (ligated and unligated) were combined and diluted for PCR analysis. Primers R2-F1-lig and R1-f2 (Supplementary Data 1) were used to amplify the cDNA mixture using the Phusion High-Fidelity PCR Master Mix with HF Buffer. The PCR product was diluted and used in a second PCR using TruSeq-i7-UDI000# and TruSeq-i5-UDI000# primers (Supplementary Data 1). Different UDIs were used to identify different replicates if they were sequenced simultaneously. The final PCR products were purified by agarose gel electrophoresis using the Zymoclean Gel DNA Recovery Kit (Zymo Research). DNA concentration was measured by real-time PCR (StepOnePlus, Thermo Fisher) using the NEBNext Library Quant Kit for Illumina (NEB) and analyzed using Illumina NovaSeq or MiSeq by the Sequencing Section at OIST.

### Sequencing data analysis

Custom Python scripts were used to analyze the sequencing data. Each read in the FASTQ file was sorted into ligated or unligated pools based on the barcode sequence. Then, the read was scanned to search for the variable catalytic core region, which was then quality-filtered to obtain all base calls with QS ≥ 20. For the F1*U^m^ and WT/Mut libraries, a maximum of one base call in the variable region was allowed to have QS < 20. For each variant, the read count of the ligated sequence (*N*_ligated_) and that of the unligated sequence (*N*_unligated_) was determined to calculate the FL (FL = *N*_ligated_/(*N*_ligated_ + *N*_unligated_)). The FL for each variant was divided by that of the WT, which was included in every generation, to calculate the RA. Each generation was assayed in duplicate, and variants were discarded if the total read count (*N*_ligated_ + *N*_unligated_) in either replicate was below 30 for the F1*U^m^ library or below 100 for all other libraries. The mean RA was calculated from the two measurements for each variant and is referred to as the RA for subsequent analysis.

### PAGE analysis of individual ligase ribozymes

DNA templates for individual ribozyme variants were constructed by annealing and extending two oligonucleotides using OneTaq 2X Master Mix with Standard Buffer (NEB). (Supplementary Data 1) The PCR products were column-purified using DNA Clean & Concentrator-5 and then transcribed in vitro as described above. Ligation reactions were performed as described above, except for the use of excess ligase ribozyme (2 μM) over the FAM-labeled substrate (FAM-F1*subA, 0.1 μM, FASMAC). Polyacrylamide gels were imaged using a Typhoon FLA9500 (GE Healthcare) and quantified using ImageJ 2.3.0 software.

### In silico selection, mutation, and recombination

Our evolutionary pipeline consisted of iterative cycles of oligo pool synthesis, experimental assay, in silico selection, in silico recombination, and in silico mutation. Flowcharts describing the steps in the algorithm are shown in Supplementary Figs. 3 and 4. Tournament selection was used as a selection method. First, a predetermined number of variants from the population were randomly selected, and the variant with the highest RA was retained as a parent. The remaining variants (losers) were returned to the population, and the process was repeated until a predetermined number of variants were selected as parents. Tournament selection allows a small percentage of medium to low RA variants to be selected along with high RA variants for the next generation. This mechanism potentially accounts for the epistatic nature of the fitness landscape, where less-active mutants might become more active later with additional mutations.

In the first design of the algorithm, two parental sequences were picked at random to be recombined using one-point crossover at a random position, and one of the resulting recombinants was randomly selected for substitution. Each position in the sequence has a 1/35 chance of mutating to one of the three other bases with an equal probability. Therefore, on average, each recombined mutant had one substitution. This process was repeated until the total number of offspring was reached. Mutants were selected only if they had not been previously selected. Finally, some mutants were randomly replaced with the controls (Supplementary Fig. 3). This strategy was used to design generations 2–5.

From generation 6 onward, parents were picked again by tournament selection. A set of pure recombinants was then generated from the parents. Next, a random variant was selected from the pool of recombinants and parents. This variant was then randomly mutated, and the process was repeated to create another set of point mutants that were generated by random substitution of parents or recombinants. The new generation consisted of a combination of pure recombinants and random point mutants (Supplementary Fig. 4).

The parameters used in the in silico algorithm, including tournament size, number of parents, number of pure recombinants, number of random mutants, and total population size, are given in Supplementary Table 1. From generation 3 onward, we increased the total population size to increase the chance of finding neutral mutants during each round of experimental screening. From generation 7 onward, we reduced the tournament size and increased the number of selected parents to account for the increased fraction of neutral mutants. This led to an overall reduction in selection stringency to ensure that some variants with lower activity were still being selected.

### Machine learning

Five machine-learning models for binary classification were trained using data from generations 1–6. All models made predictions by trying to fit a function that describes the relationship between the input features, which in this case is the position and identity of the nucleotide in each ribozyme sequence, and the class labels that are either neutral or deleterious. For a more comprehensive discussion of different machine-learning techniques, we refer the reader to the review in ref. 55, and each model is also briefly described below.

LR is a linear model that assigns different weights to the input features. Predictions are made using a linear combination of the input features and their weights, followed by a sigmoid function that outputs the class probability. LR can only model the additive contribution of each mutation to fitness and therefore cannot model nonlinear interactions between positions.

An SVM with a linear kernel assumes that the classes in the data are linearly separable in the feature space and attempts to draw a boundary line to separate them. The optimal solution was achieved by maximizing the distance between each class and the boundary line. SVM with a linear kernel can only model a linear combination of mutations, similar to LR, although the tendency to overfit SVM is lower. Overfitting is observed when a model accurately predicts the training data but creates poor predictions for new data points.

The k-nearest neighbor (k-NN) method does not assume a linear separation of classes. The prediction for a new input is made based on the majority class of the *k* number of neighboring training points closest to the new input in the feature space. This allows k-NN to model nonlinearity better than SVM or LR, but it is more affected by noise in the data and is more likely to become overfit.

The GBDT makes predictions by constructing a group of “trees” that branch out each time a condition for a feature is met (e.g., is position 23 in the sequence a guanosine?). The tree depth determines the complexity of these conditions for making the final decision regarding the class label. Gradient boosting is a technique that uses a large number of trees with shallow depths to vote on the final class label. This typically enables a higher prediction accuracy and less overfitting than individual trees or a small group of very deep trees. The GBDT can model more complex nonlinear interactions than LR and SVM.

The MLP is a simple neural network model. A neural network consists of a group of individual “neurons” that take an input value and transform them using a nonlinear function. These neurons are arranged in fully connected layers, meaning that the output of one neuron becomes the input of the other neuron. This architecture allows a neural network to approximate any function. This means that MLP can potentially model mutational interactions or epistasis at a very high order better than the other models. However, neural networks require substantially more data to accurately learn a function without overfitting.

For training, the sequences were one-hot encoded and flattened into 1 × 140 binary vectors. Thirty percent of the dataset was used as the testing set, and the rest was used as the training set. The LR, k-NN, SVM, and GBDT were trained using the Python scikit-learn package. k-NN, LR, and SVM were trained using the default hyperparameters for the binary classification of sequences into neutral (RA ≥ 0.2) or deleterious (RA < 0.2). The GBDT was trained in the same manner using a maximum tree depth of 10. The MLP was written using the TensorFlow 2 Python library. The model consisted of three dense layers with rectified linear unit (ReLU) activation, batch normalization, and 20% dropout. The dense layers consisted of 128, 64, and 32 neurons, respectively. This was followed by a final dense layer with sigmoid activation for the classification output. The model was compiled using the Adam optimizer, with a learning rate of 0.005. Binary cross-entropy was used as the loss function. During training, 10% of the training set was used as a validation set, and the model was trained for 100 epochs with a batch size of 1024. All the trained model performances were evaluated on the test dataset using precision and recall as metrics. All codes were written in Python 3.9. The software libraries used were pandas 1.4.4, numpy 1.21.2, tqdm 4.62.3, scipy 1.7.3, matplotlib 3.5.1, seaborn 0.11.2, scikit-learn 1.0.2 and tensorflow 2.8.0.

### Evolutionary algorithm

The MLP model was retrained using data from generations 1–7 in the same manner as described for Machine learning. Model performances were also tested using tenfold cross-validation (Supplementary Fig. 9). To account for class imbalance, we adjusted the prediction threshold using the receiver operating characteristic (ROC) curve. Prediction thresholds that produced the largest geometric mean (${{\mbox{Geometric mean }}}=\sqrt{{{\mbox{True positive rates}}}\times {{\mbox{(}}}1{{\mbox{-False positive rates)}}}}$)were used for subsequent classification by the model. Generation 7 was used as the starting parent population for the in silico evolution. Tournament selection was used to select variants as parents. If more than one variant in the tournament was classified as neutral, then a random variant was selected. In each generation, 80% of the variants were created by recombination, and the remainder were created by point mutations. These variants were classified as neutral or deleterious mutants using MLP (Supplementary Fig. 5). This was repeated over 100 rounds, and the average Hamming distance in each round was tracked to ensure an increase in diversity (Supplementary Fig. 10). After 100 rounds of evolution, the mean Hamming distance plateaued at ~13. Increasing the number of rounds of in silico evolution might lead to an increased average Hamming distance; however, this was slowed by the increased search space and a likely increase in false positives. For the last round, the total number of variants was increased to 12,000 to maximize the coverage of the sequence space for experimental screening, and only variants that were predicted to be neutral by the MLP were selected as generation 8. The parameters of the in silico evolutionary algorithm are listed in Supplementary Table 1.

### Expected RA and robustness calculation

In a log-additive model, the expected RA (expRA) of mutant $g$ with no epistasis was calculated as ${{{{{\rm{ln}}}}}}\left({{{\exp }}{{{{{\rm{RA}}}}}}}_{g}\right)={\sum }_{i}^{m}{{{{{\rm{ln}}}}}}({M}_{i}){\gamma }_{i}$, where $m=105$ is the total number of single mutants and ${M}_{i}$ is the RA of a single mutant. ${\gamma }_{i}$ = 1 if the sequence contains the mutation ${M}_{i}$ and ${\gamma }_{i}$ = 0 if it does not. The coefficient of determination (*R*^2^) was used to determine the fraction of the observed ln(RA) predicted by the model. *R*^2^ was calculated using the r2_score function in the Python scikit-learn package using default parameters.

Equation $\omega (n)={e}^{-\alpha {n}^{\beta }}$ was fitted using the nonlinear least-squares curve fitting function in the SciPy Python library. *ω(n)* is the fraction of neutral mutants (RA ≥ 0.2) at Hamming distance *n* from the reference sequence (WT or F1*U^m^). *α* is the decay parameter, where a lower *α* indicates higher mutational robustness. *β* is the strength of directional epitasis. When *β* > 1, there is an excess of negative epistasis; when *β* < 1 there is an excess of positive epistasis. If *β* is equal to one, there is a balanced mix of positive and negative epistasis, or there is no epistasis.

### Estimation of the fraction of reciprocal sign epistasis

For generations 1–8 and the WT/Mut library, we separately identified all unique pairs of mutants that differed by two substitutions. Of these, pairs of mutants in which both intermediate single mutants were present in the dataset were retained. For each pair of sequences, reciprocal sign epistasis was identified if both the RA values were higher or lower than those of the intermediate single mutants. For each set of sequences, the sequence with the lowest Hamming distance to the WT was used as the reference sequence. This was only used to measure reciprocal sign epistasis at each mutational step (Hamming distance) from the WT. The identification of reciprocal sign epistasis is not affected by the choice of the reference sequence. The fraction of reciprocal sign epistasis was calculated by dividing the number of reciprocal sign epistasis by the total number of 2^2^ genotype subgraphs identified at each Hamming distance from the WT.

### Analyzing epistasis of a combinatorially complete landscape

A combinatorially complete landscape consists of 2^N^ possible variants. In the case of the WT/Mut library, this equaled 65,336 possible combinations for 16 mutations (2^16^). Each variant can be represented as a 16-bit binary with 1 or 0 as each digit, indicating the presence or absence of each mutation. The ln(RA) value of each variant can be sorted according to the binary order to give vector **w**. Vector **w** can be linearly mapped into the epistatic terms relative to a single reference *e*_rel_ using ${e}_{{{{{{\rm{rel}}}}}}}={{{{{\bf{Gw}}}}}}$. **G** is a matrix that defines all interactions from order 0 to *n* and can be recursively defined as:

1 $${{{{{{\bf{G}}}}}}}_{n+1}=\left(\begin{array}{cc}{{{{{{\bf{G}}}}}}}_{n} & 0\\ {-{{{{{\bf{G}}}}}}}_{n} & {{{{{{\bf{G}}}}}}}_{n}\end{array}\right){{{{{\rm{with}}}}}}\;{{{{{{\bf{G}}}}}}}_{0}=1$$

To calculate the background-averaged interaction terms, *e*_avg_ we use the equation ${e}_{{{{{{\rm{avg}}}}}}}={{{{{\bf{VHw}}}}}}$.

**H** is the Hadamard matrix which can be defined recursively as:

2 $${{{{{{\bf{H}}}}}}}_{n+1}=\left(\begin{array}{cc}{{{{{{\bf{H}}}}}}}_{n} & {{{{{{\bf{H}}}}}}}_{n}\\ {{{{{{\bf{H}}}}}}}_{n} & {-{{{{{\bf{H}}}}}}}_{n}\end{array}\right){{{{{\rm{with}}}}}}\;{{{{{{\bf{H}}}}}}}_{0}=1$$

**V** is a weighting matrix that can be defined recursively as:

3 $${{{{{{\bf{V}}}}}}}_{n+1}=\left(\begin{array}{cc}\frac{1}{2}{{{{{{\bf{V}}}}}}}_{n} & 0\\ 0 & {-{{{{{\bf{V}}}}}}}_{n}\end{array}\right){{{{{\rm{with}}}}}}\;{{{{{{\bf{V}}}}}}}_{0}=1$$

Multiplying **w** by **VH** yields the weighted Walsh–Hadamard transform of ln(RA) values. **w** can be reconstructed from *e*_rel_ or *e*_avg_ by multiplying with the inverse of the matrix **VH** or **G**. (${{{{{\bf{w}}}}}}={({{{{{\bf{VH}}}}}})}^{-1}{e}_{{{{{{\rm{avg}}}}}}},\;{{{{{\bf{w}}}}}}={{{{{{\bf{G}}}}}}}^{-1}{e}_{{{{{{\rm{rel}}}}}}}$) More detailed explanations of the theory can be found in ref. 42. Predictions made by reconstructed **w** were compared to observed **w** using *R*^2^ calculated in the same way as described in “Expected RA and robustness calculation”.

### Reporting summary

Further information on research design is available in the Nature Research Reporting Summary linked to this article.

## Supplementary information

Supplementary Information

Description of Additional Supplementary Files

Supplementary Data 1

Reporting Summary

## Data Availability

The raw sequencing reads generated and analyzed in this study have been deposited in the Sequencing Reads Archive under BioProject number PRJNA863914. Processed and filtered read files with all ribozyme sequences and their associated activity measurements are deposited in Zenodo: 10.5281/zenodo.6945203 (ref. 56).

## Code availability

All codes that reproduce all figures in this study are deposited and freely accessible from Zenodo: 10.5281/zenodo.6945203 (ref. 56).
