# Supplementary material for: Experimental exploration of a ribozyme neutral network using evolutionary algorithm and deep learning
Source: Nat Commun. 2022 Aug 17;13:4847. doi: 10.1038/s41467-022-32538-z (PMC9385714; doi:10.1038/s41467-022-32538-z)
Supplement: Supplementary file 1 — Supplementary Information [file 41467_2022_32538_MOESM1_ESM.pdf]

# Supplementary Information

## Experimental exploration of a ribozyme neutral network using evolutionary algorithm and deep learning

Rachapun Rotrattanadumrong and Yohei Yokobayashi*

Nucleic Acid Chemistry and Engineering Unit
Okinawa Institute of Science and Technology Graduate University
Onna, Okinawa, 9040495

*Corresponding author: yohei.yokobayashi@oist.jp

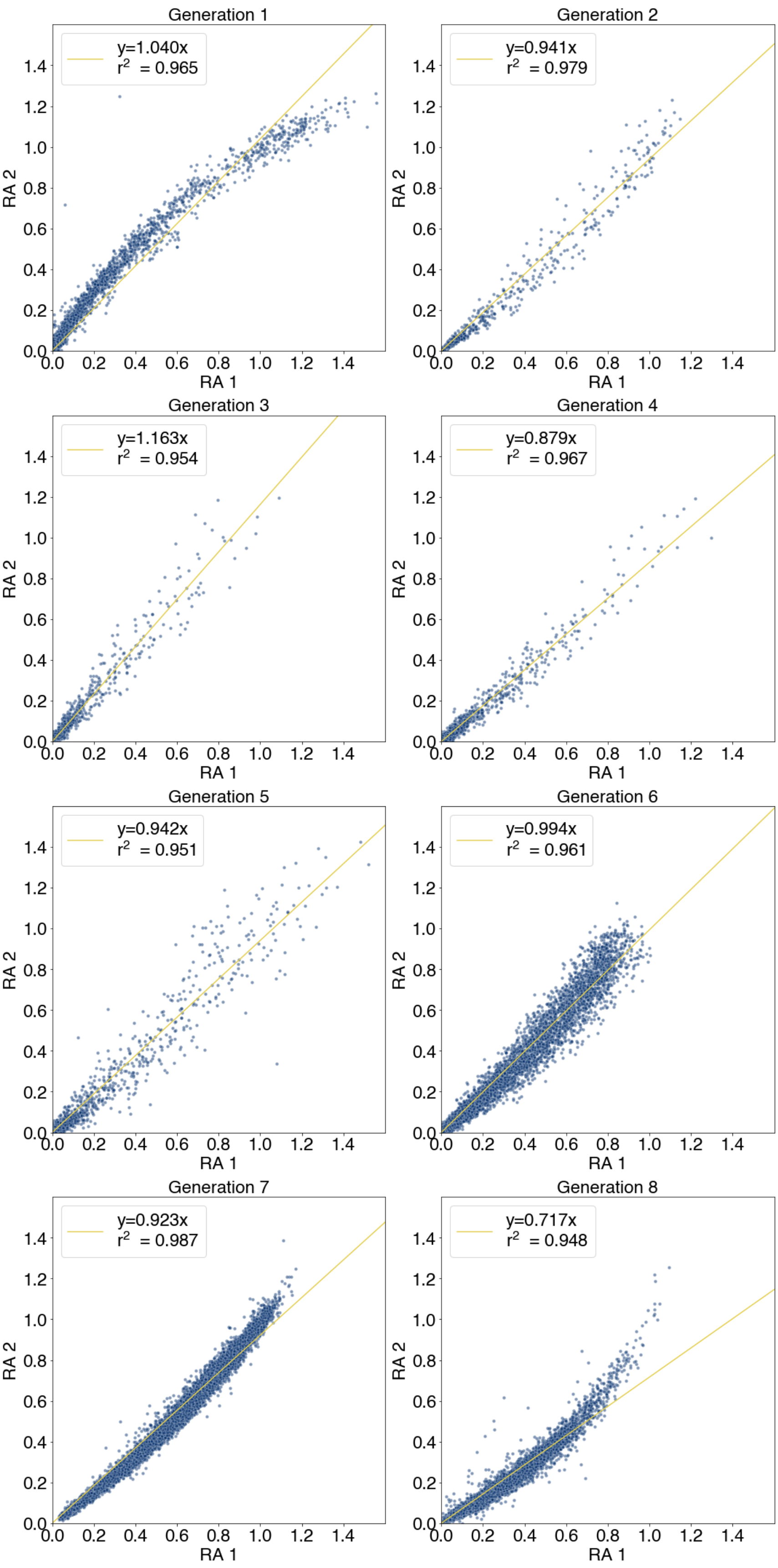


**Supplementary Figure 1: Reproducibility of RA values calculated from sequencing assays.** Two independent experimental assays of ribozyme activities were performed each generation. RA values calculated in each repeat are compared by the square of Pearson's correlation coefficient ($r^2$) values to confirm reproducibility of the sequencing experiments.

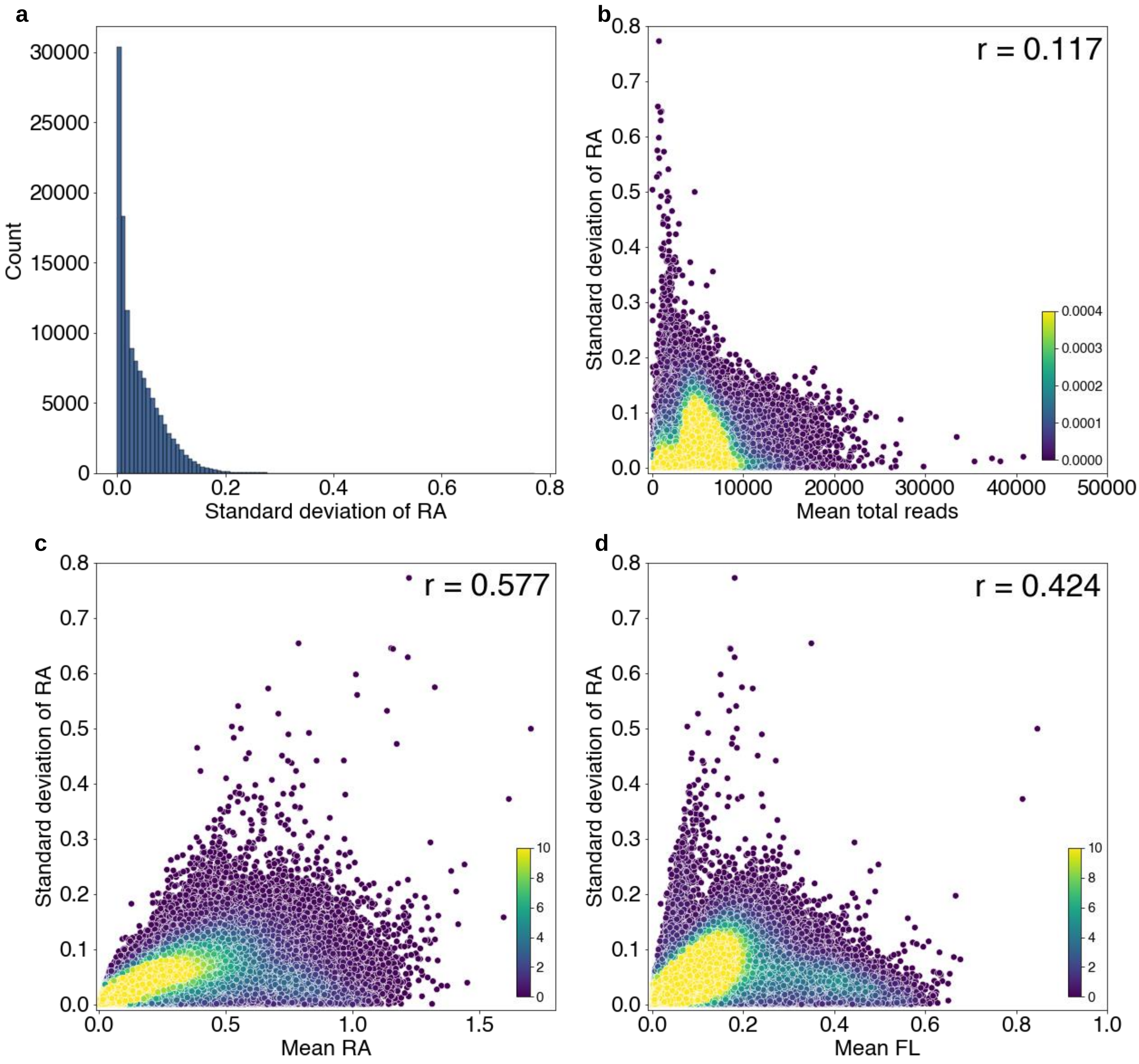


**Supplementary Figure 2: Correlation between standard deviation of RA, mean total read counts, mean RA, and mean FL. a,** Standard deviation calculated from RA values from two independent sequencing assays plotted as a histogram. Standard deviation plotted against **b,** mean total reads, **c,** mean RA, and **d,** mean FL. Color bars indicate data density at each point. Pearson's correlation coefficient (r) were calculated for all 129,507 data points after filtering for read count and sequence quality.

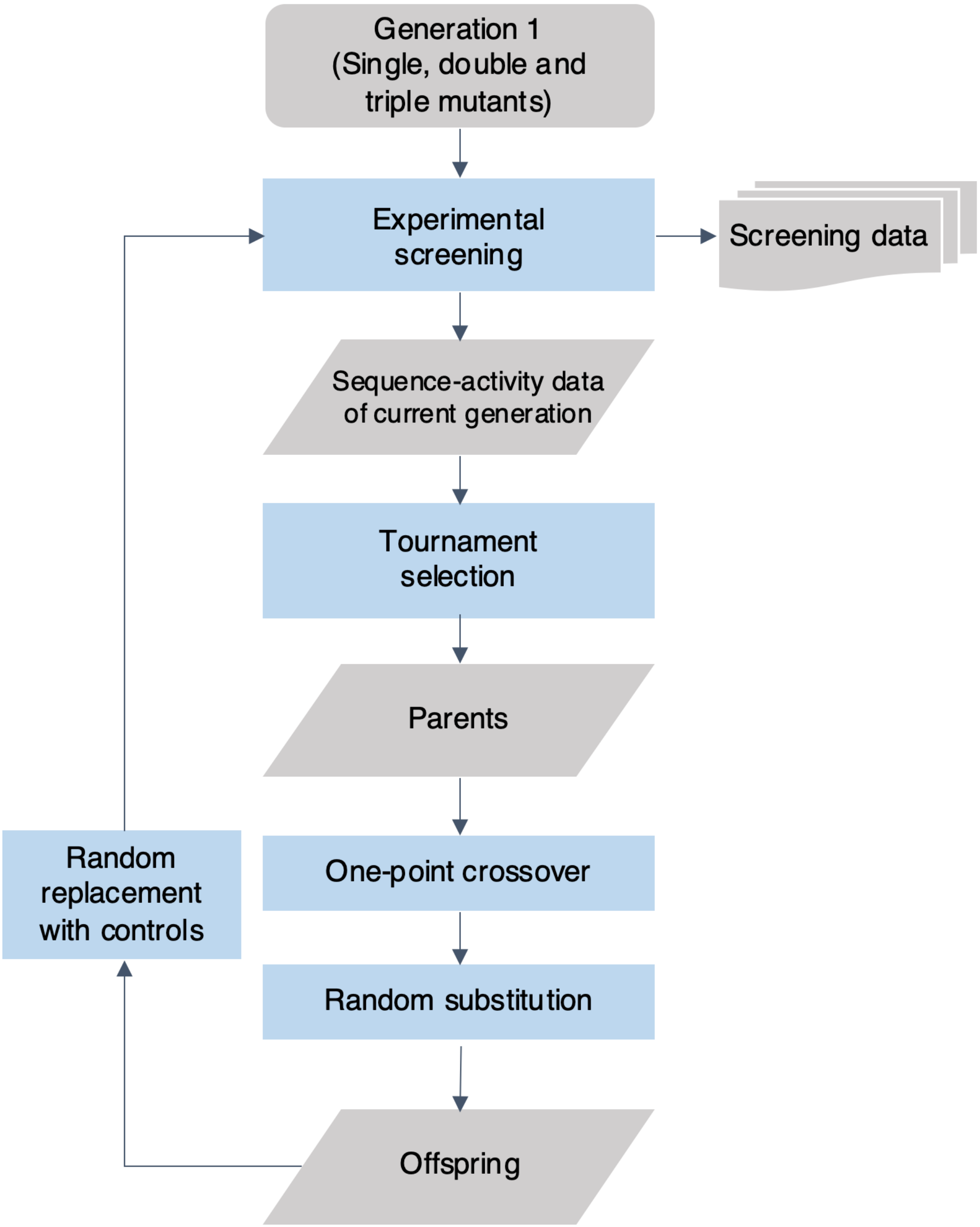


**Supplementary Figure 3: Flow-chart of the hybrid in silico/ in vitro algorithm used to design generations 2–5.**The designed sequences were experimentally assayed to generate a sequence-activity dataset. Based on the dataset, variants with higher activities were selected as parents by tournament selection. The parental sequences were used to generate offspring by one-point crossover and random substitution until the desired number of offspring sequences were obtained. Some of the sequences were randomly replaced with control sequences for normalization across generations.

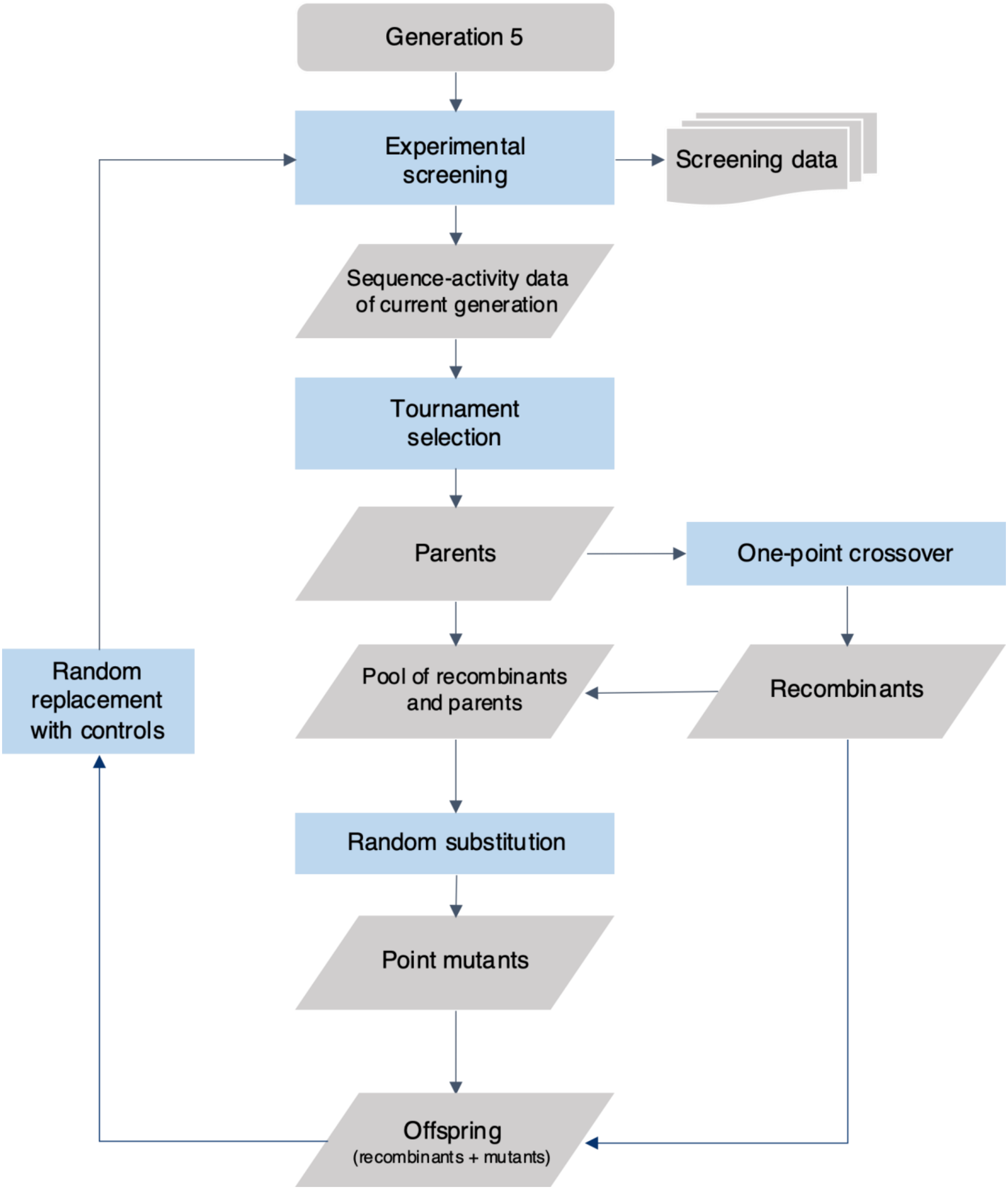


**Supplementary Figure 4: Flow-chart of the hybrid in silico/ in vitro algorithm used to design generations 6 and 7.** The algorithm was updated based on the performance observed in generations 2-5. In this algorithm, tournament selection was still used to select parental sequences based on experimentally determined activity. However, offspring sequences were generated from a combination of parents that underwent only one-point crossover (recombinants), and parents or recombinants that also underwent random substitution (point mutants). Some of the sequences were randomly replaced with control sequences for normalization across generations.

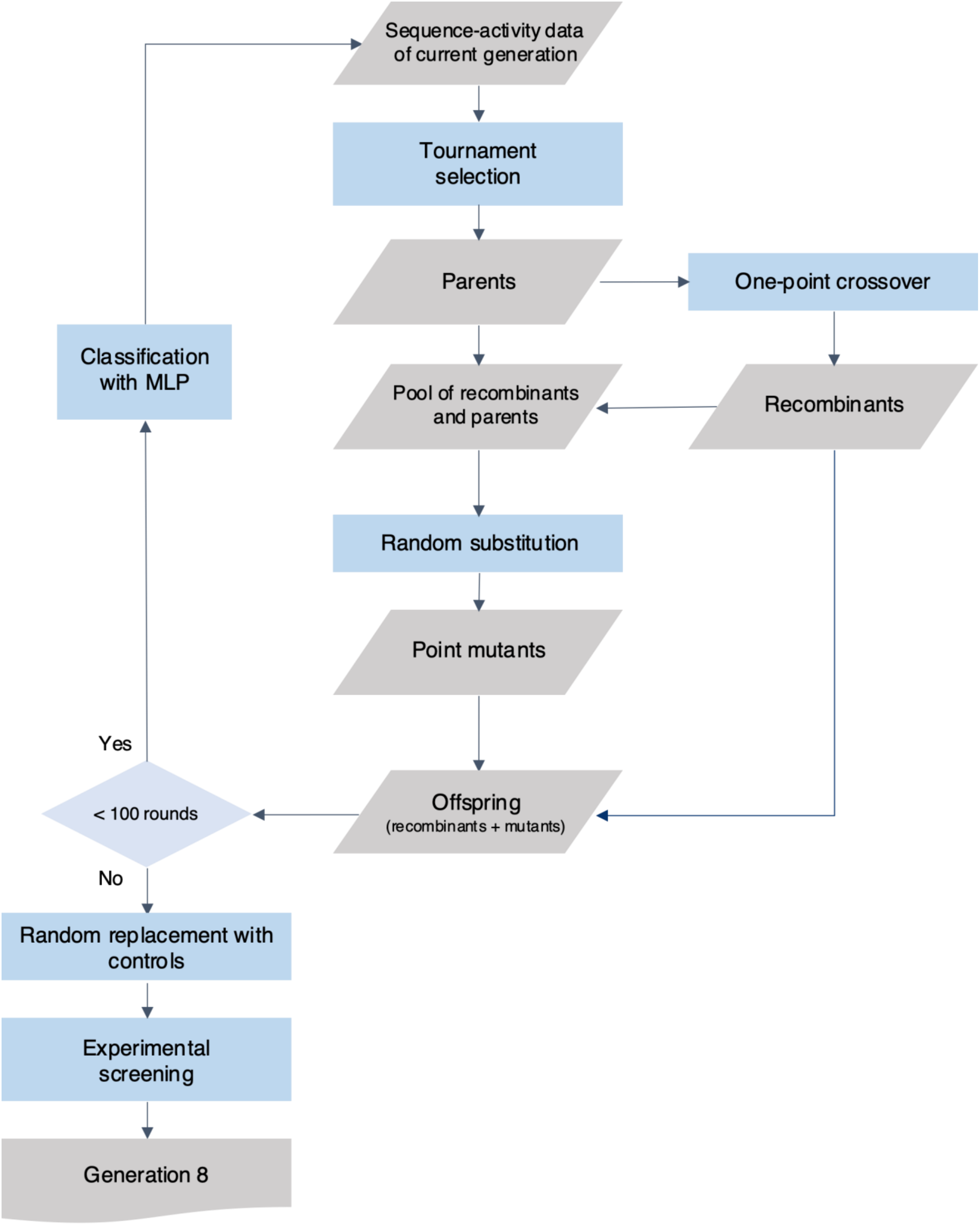


**Supplementary Figure 5: Flow-chart of the completely in silico evolutionary algorithm used to design generation 8.** In this algorithm, the process of selection, recombination and mutations are identical to those used to design generations 6 and 7. However, the experimental assay was replaced by computational classification using a multilayer perceptron model (MLP) trained with data from generations1-7. Starting with generation 7, selection, recombination, mutation, and MLP classification were performed for 100 rounds. Some of the sequences were randomly replaced with control sequences for normalization across generations, and the sequences were experimentally assayed to yield the generation 8 dataset.

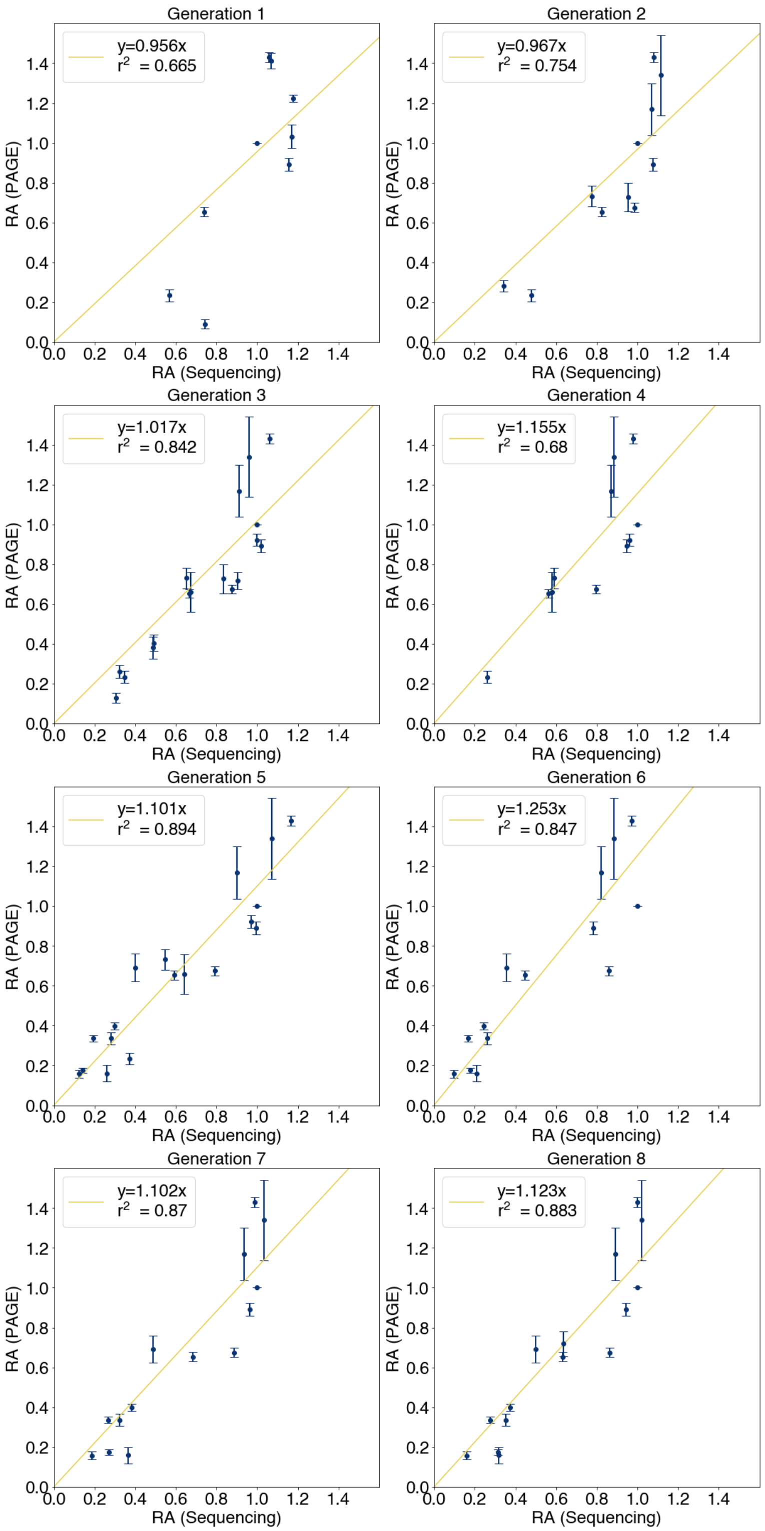


**Supplementary Figure 6: Correlation of RA values measured by sequencing and PAGE assays.** Some variants in each generation with varying activities were individually assayed by PAGE. An excess of the ribozyme was reacted with the fluorescently labeled substrate (FAM-F1*subA). The ligated and unligated substrates were separated by PAGE and imaged to calculate RA. The WT was included in every experiment as a standard. Data are presented as mean values +/− SD with n = 3. Square of Pearson's correlation coefficient ($r^2$) measures correlation between RA values determined by the two methods.

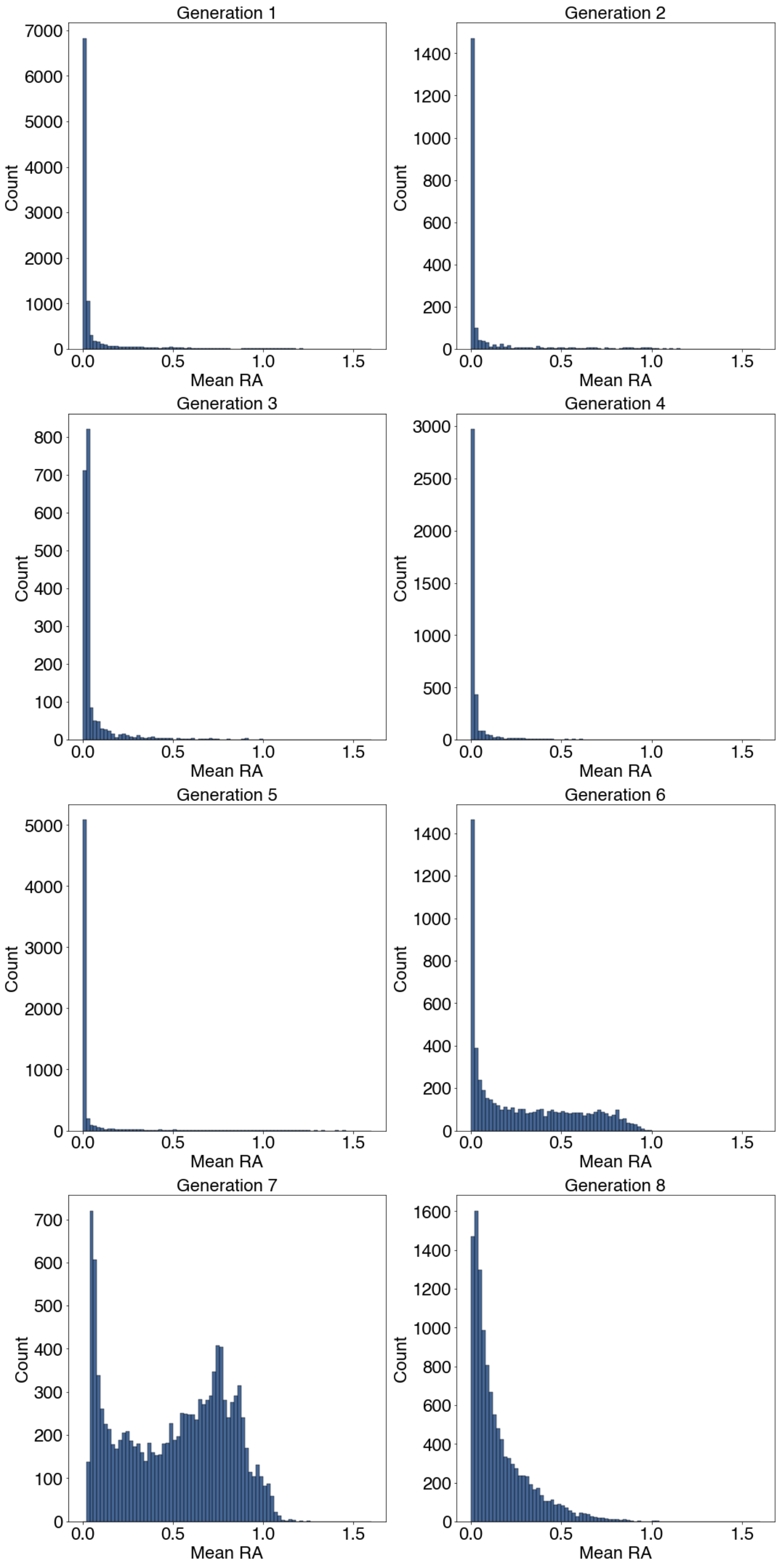


**Supplementary Figure 7: Histograms showing distribution of fitness (RA) in each generation of ribozyme population.** The variants in each generation were divided into 80 discrete bins between 0 and 1.6 (mean RA) with a bin width of 0.02.

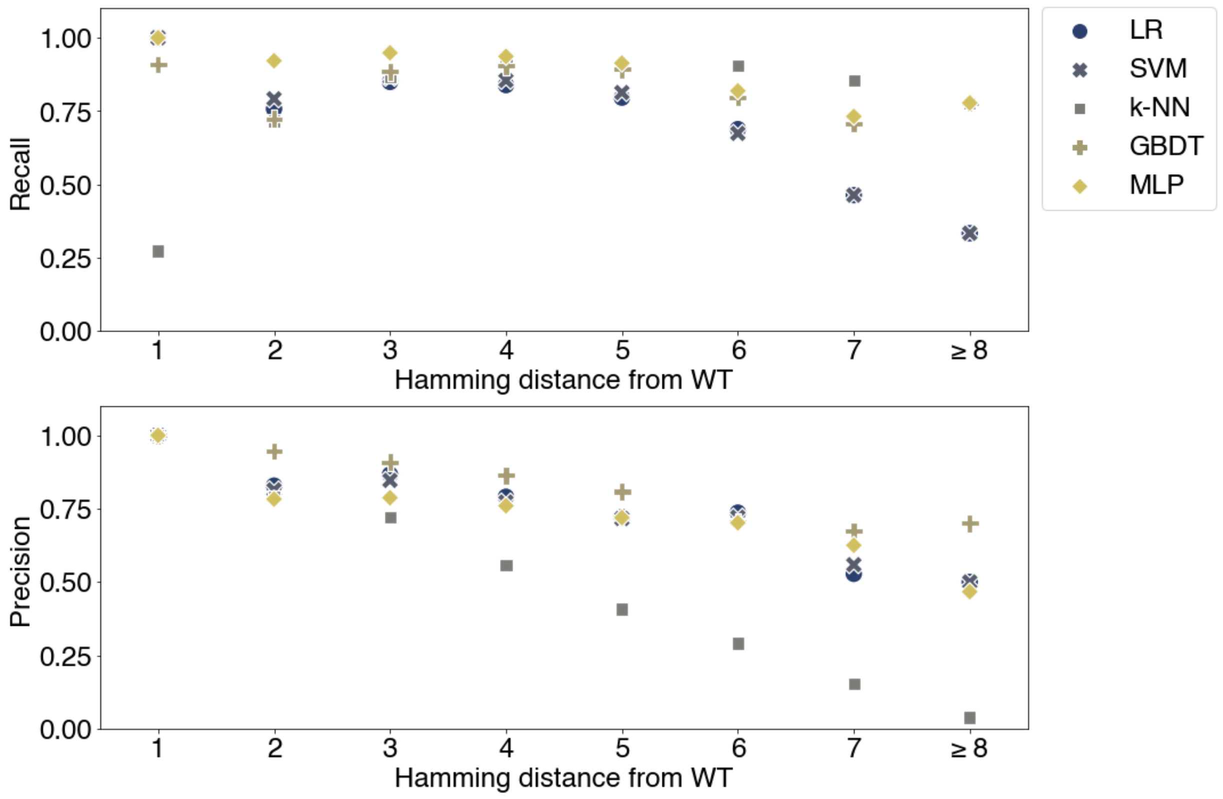


**Supplementary Figure 8: Dependence of precision and recall of the machine-learning models on the Hamming distance from the WT.** The testing dataset was used to generate the plot.

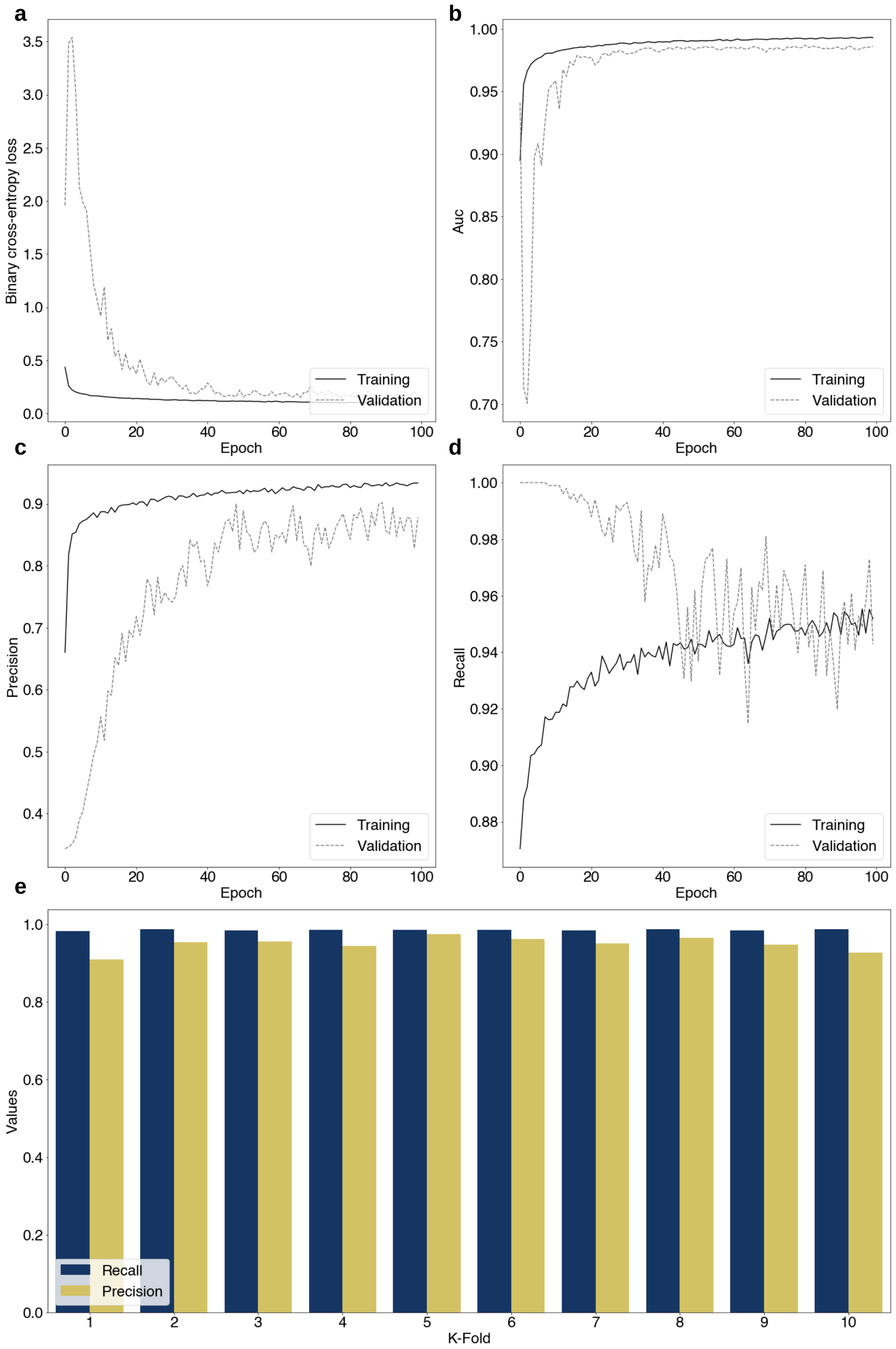


**Supplementary Figure 9: Multilayer perceptron (MLP) model training and testing.** Training history using data from generations 1-7. A total of 26,374 variants were used for training and 2,930 variants were used for validation at the end of each epoch. Trainings were conducted for 100 epochs tracked by **a,** binary cross-entropy loss, **b,** area under curve (auc), **c,** precision, and **d,** recall. **e,** Precision and recall of each fold during 10-fold cross validation on a total of 41,863 variants from generations 1–7.

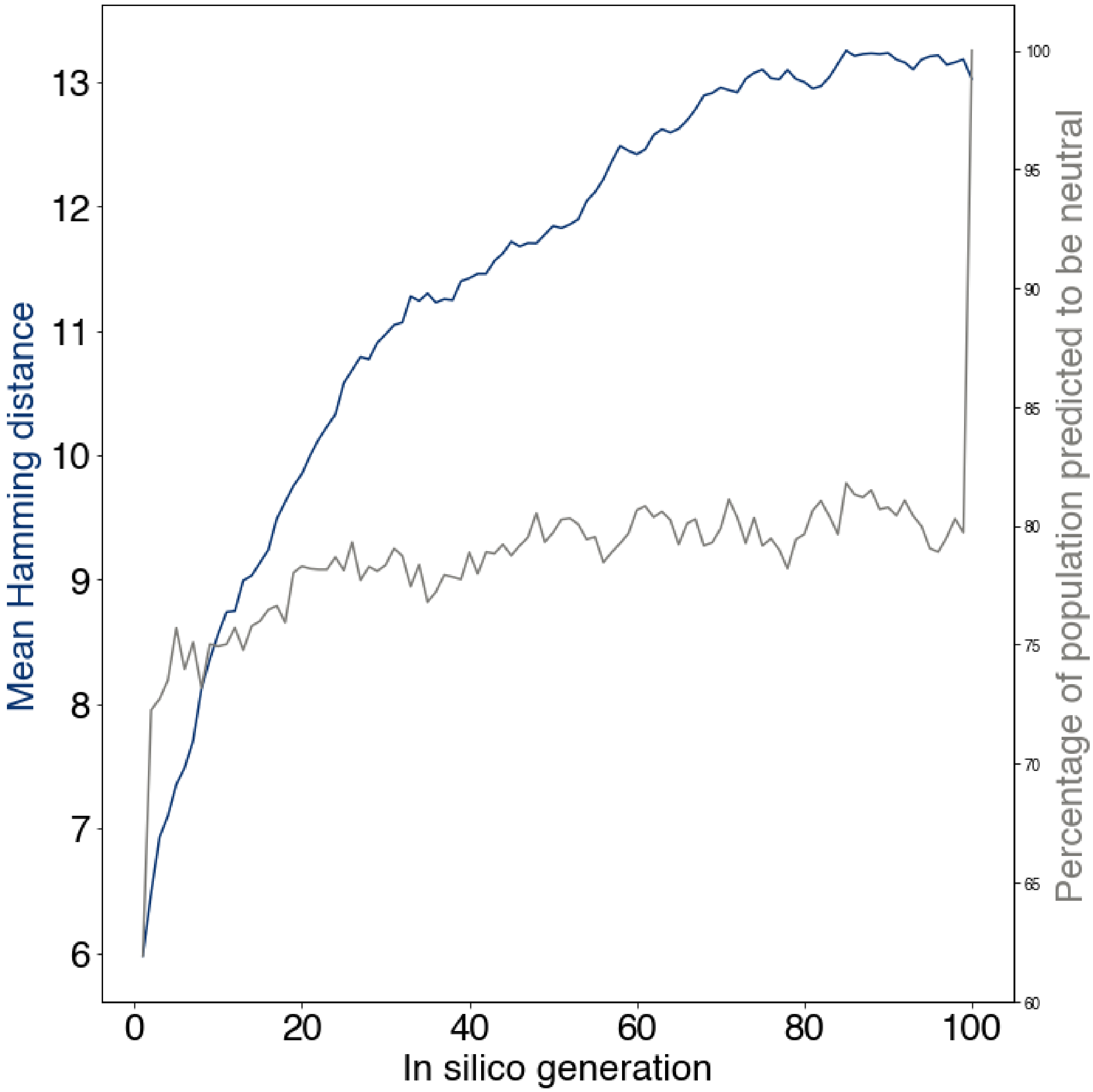


**Supplementary Figure 10: In silico evolution of generation 8.** The mean Hamming distance and percentage of the population predicted to be neutral by MLP calculated at each generation during in silico evolution. In generation 100, the variants were picked only if predicted to be neutral by the MLP.

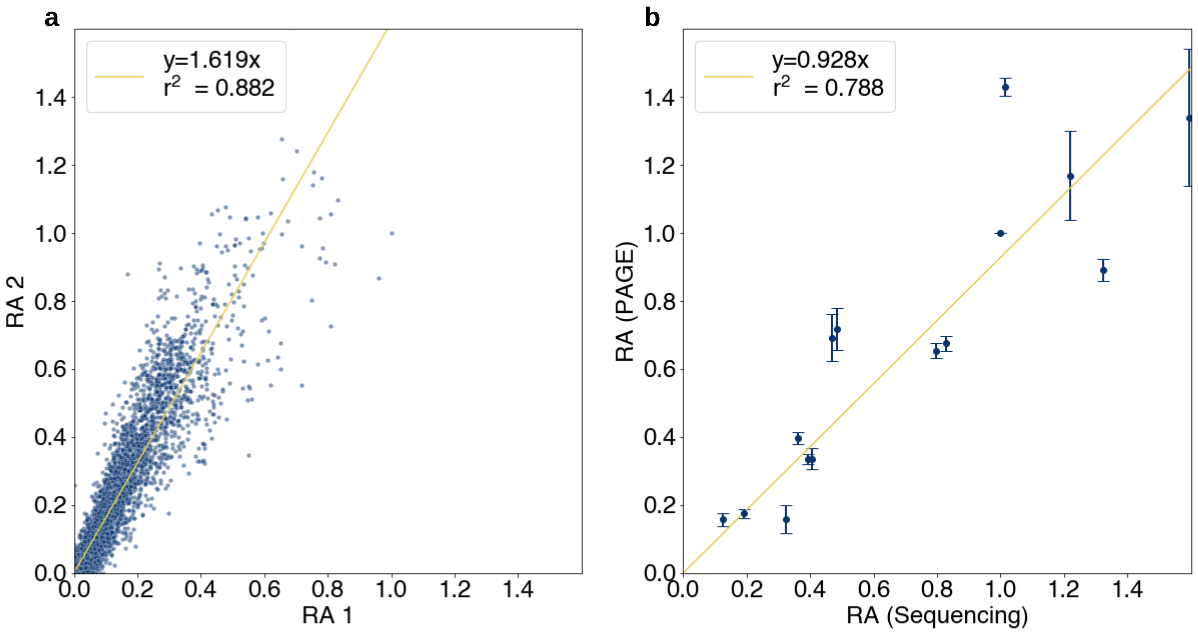


**Supplementary Figure 11: Reproducibility of F1*U$^m$ local landscape library. a,** Reproducibility of RA values calculated by two independent sequencing experiments. **b,** Correlation between RA from sequencing and PAGE assays. Data are presented as mean values +/− SD with n = 3.

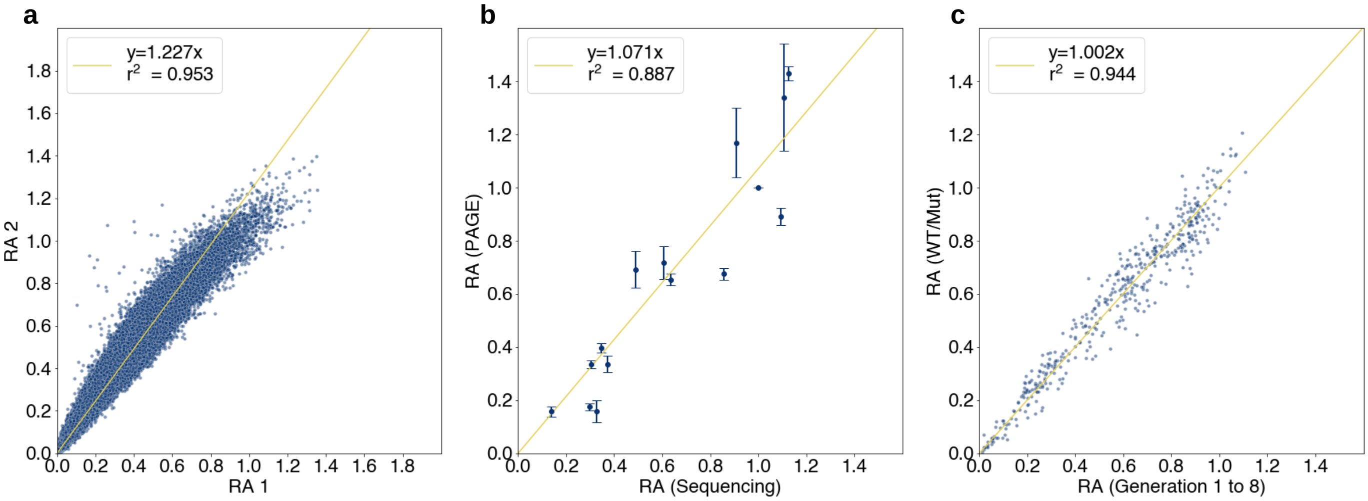


**Supplementary Figure 12: Reproducibility of WT/Mut landscape library. a,** Reproducibility of RA calculated by two independent sequencing experiments. **b,** Correlation between RA values from sequencing and PAGE assays. Data are presented as mean values +/− SD with n = 3. **c,** RA values of 441 variants screened in both WT/Mut library and generations 1–8.

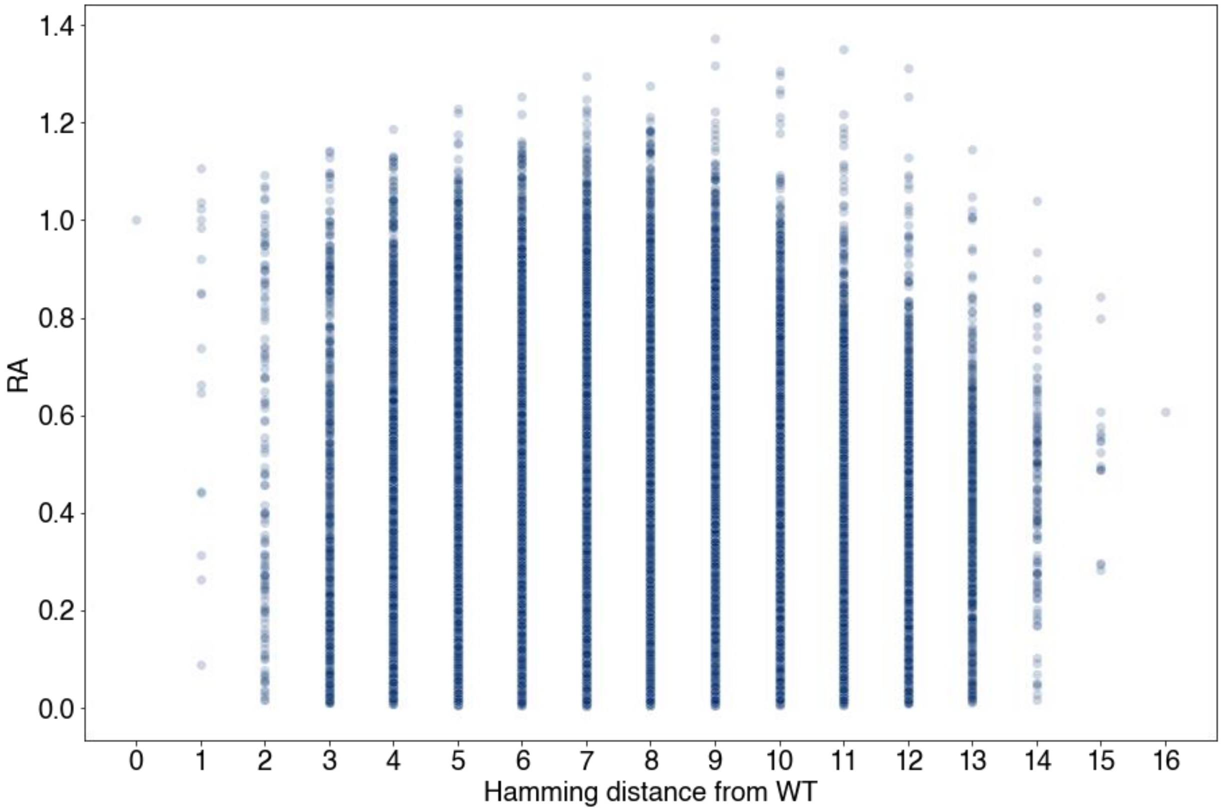


**Supplementary Figure 13: The combinatorial landscape between F1*U and F1*U$^{m}$ (WT/Mut).** The RA values of each variant plotted according to their Hamming distance from the WT.

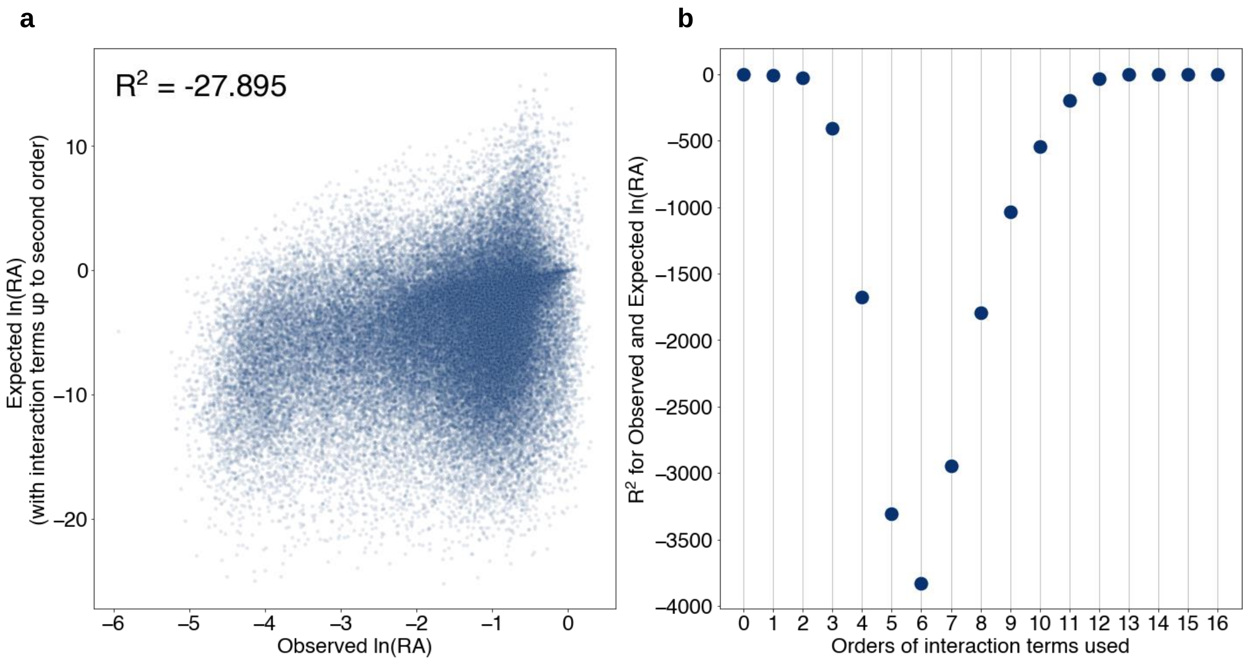


**Supplementary Figure 14: Correlation of the observed WT/Mut landscape and the predicted landscape using relative epistatic model with WT as reference background. a,** The observed ln(RA) values of WT/Mut library and the expected ln(RA) values from a relative epistatic model that only include the first- and second-order epistatic terms. **b,** The coefficients of determination ($R^2$) values between the observed ln(RA) and the expected ln(RA). Expected ln(RA) were calculated at each step by cumulatively adding WT-relative epistatic terms of successively higher order. For each step, $R^2$ scores were calculated using all variants in the library.

**Supplementary Table 1:** Parameters used in the evolutionary algorithm to design each generation of the ligase ribozymes.

| Generation | Tournament size | Number of parents | Number of pure recombinants | Number of mutants | Population size (Number of offspring) |
|---|---|---|---|---|---|
| 1 | N/A | N/A | N/A | N/A | 10000 |
| 2 | 300 | 200 | N/A | N/A | 2000 |
| 3 | 300 | 200 | N/A | N/A | 2000 |
| 4 | 300 | 200 | N/A | N/A | 4000 |
| 5 | 300 | 200 | N/A | N/A | 6000 |
| 6 | 300 | 200 | 4000 | 2000 | 6000 |
| 7a | 50 | 1000 | 8000 | 2000 | 10000 |
| 7b | 50 | 1000 | 800 | 200 | 1000 |
| 7c | Generated by shuffling generation 7b | | | | 1000 |
| In silico evolution | 32 | 1000 | 4800 | 1200 | 6000 |
| 8 | 32 | 1000 | 9600 | 2400 | 12000 |

**Supplementary Table 2:** The number of all $2^2$ subgraphs identified in generations 1–8 and WT/Mut library. Each subgraph consists of a pair of mutants that differ by two mutations and their constituent single mutants. The subgraphs are categorized according to the Hamming distance of the reference sequence to the wild-type (WT).

| Hamming distance to WT | Generation 1 to 8 | WT/Mut |
|---|---|---|
| 0 | 10710 | 240 |
| 1 | 64098 | 3360 |
| 2 | 54312 | 21840 |
| 3 | 47164 | 87360 |
| 4 | 29450 | 240240 |
| 5 | 7002 | 480480 |
| 6 | 814 | 720720 |
| 7 | 76 | 823680 |
| 8 | 20 | 720720 |
| 9 | 20 | 480480 |
| 10 | 54 | 240240 |
| 11 | 78 | 87360 |
| 12 | 86 | 21840 |
| 13 | 84 | 3360 |
| 14 | 64 | 240 |
| 15 | 26 | 0 |
| 16 | 10 | 0 |

**Supplementary Table 3:** The coefficients of determination ($R^2$) values between the observed ln(RA) and the expected ln(RA) of the WT/Mut library. Expected ln(RA) values were calculated for all variants in the library by cumulatively adding background-averaged epistatic terms of successively higher order. Only variants with Hamming distance higher than the maximum order of epistatic terms included at each step were used to calculate $R^2$ score. $R^2$ cannot be reliably calculated with less than two samples, therefore it was not calculated for order ≤ 15.

| Included epistatic terms (order ≤ ) | Included mutants (HD ≥ ) | $R^2$ | Number of mutants analysed |
|---|---|---|---|
| 0 | 1 | $-5.13\times10^{-10}$ | 65535 |
| 1 | 2 | 0.190 | 65519 |
| 2 | 3 | 0.540 | 65399 |
| 3 | 4 | 0.728 | 64839 |
| 4 | 5 | 0.854 | 63019 |
| 5 | 6 | 0.922 | 58651 |
| 6 | 7 | 0.961 | 50643 |
| 7 | 8 | 0.980 | 39203 |
| 8 | 9 | 0.991 | 26333 |
| 9 | 10 | 0.996 | 14893 |
| 10 | 11 | 0.998 | 6885 |
| 11 | 12 | 0.999 | 2517 |
| 12 | 13 | 1.000 | 697 |
| 13 | 14 | 1.000 | 137 |
| 14 | 15 | 1.000 | 17 |
| 15 | 16 | N/A | 1 |
