# Supplementary material for: Experimental exploration of a ribozyme neutral network using evolutionary algorithm and deep learning
Source: Nat Commun. 2022 Aug 17;13:4847. doi: 10.1038/s41467-022-32538-z (PMC9385714; doi:10.1038/s41467-022-32538-z)
Supplement: Supplementary file 2 — Description of Additional Supplementary Files [file 41467_2022_32538_MOESM2_ESM.pdf]

Description of Additional Supplementary Files

Supplementary Data 1: Primers and oligonucleotides sequences for the construction of ribozyme library and individual ribozyme mutants.
