# Supplementary material for: Experimental exploration of a ribozyme neutral network using evolutionary algorithm and deep learning
Source: Nat Commun. 2022 Aug 17;13:4847. doi: 10.1038/s41467-022-32538-z (PMC9385714; doi:10.1038/s41467-022-32538-z)
Supplement: Supplementary file 4 — Reporting Summary [file 41467_2022_32538_MOESM4_ESM.pdf]

nature portfolio

Corresponding author(s): Yohei Yokobayashi

Last updated by author(s): Jul 30, 2022

Reporting Summary

Nature Portfolio wishes to improve the reproducibility of the work that we publish. This form provides structure for consistency and transparency in reporting. For further information on Nature Portfolio policies, see our Editorial Policies and the Editorial Policy Checklist.

Statistics

For all statistical analyses, confirm that the following items are present in the figure legend, table legend, main text, or Methods section.

n/a	Confirmed	
☐	☒	The exact sample size (*n*) for each experimental group/condition, given as a discrete number and unit of measurement
☐	☒	A statement on whether measurements were taken from distinct samples or whether the same sample was measured repeatedly
☒	☐	The statistical test(s) used AND whether they are one- or two-sided<br>*Only common tests should be described solely by name; describe more complex techniques in the Methods section.*
☒	☐	A description of all covariates tested
☐	☒	A description of any assumptions or corrections, such as tests of normality and adjustment for multiple comparisons
☐	☒	A full description of the statistical parameters including central tendency (e.g. means) or other basic estimates (e.g. regression coefficient) AND variation (e.g. standard deviation) or associated estimates of uncertainty (e.g. confidence intervals)
☒	☐	For null hypothesis testing, the test statistic (e.g. *F*, *t*, *r*) with confidence intervals, effect sizes, degrees of freedom and *P* value noted<br>*Give P values as exact values whenever suitable.*
☒	☐	For Bayesian analysis, information on the choice of priors and Markov chain Monte Carlo settings
☒	☐	For hierarchical and complex designs, identification of the appropriate level for tests and full reporting of outcomes
☐	☒	Estimates of effect sizes (e.g. Cohen's *d*, Pearson's *r*), indicating how they were calculated

Our web collection on statistics for biologists contains articles on many of the points above.

Software and code

Policy information about availability of computer code

Data collection	No software was used for data collection.
Data analysis	Raw single-end sequencing reads were quality filtered using a custom Python script as described in Methods. All analyses were performed using Python 3.9. The software libraries used were pandas 1.4.4, numpy 1.21.2, tqdm 4.62.3, scipy 1.7.3, matplotlib 3.5.1, seaborn 0.11.2, scikit-learn 1.0.2 and tensorflow 2.8.0. All codes are available as Jupyter notebooks deposited in Zenodo (https://doi.org/10.5281/zenodo.6945203) PAGE analysis of individual ribozymes were performed using ImageJ 2.3.0. Prediction of ribozyme secondary structures was performed using ViennaRNA 2.5.1.

For manuscripts utilizing custom algorithms or software that are central to the research but not yet described in published literature, software must be made available to editors and reviewers. We strongly encourage code deposition in a community repository (e.g. GitHub). See the Nature Portfolio guidelines for submitting code & software for further information.

Data

Policy information about availability of data

All manuscripts must include a data availability statement. This statement should provide the following information, where applicable:

- Accession codes, unique identifiers, or web links for publicly available datasets
- A description of any restrictions on data availability
- For clinical datasets or third party data, please ensure that the statement adheres to our policy

The raw sequencing reads generated in this study have been deposited in the Sequencing Reads Archive (https://www.ncbi.nlm.nih.gov/bioproject/PRJNA863914). Processed and filtered read files are deposited in Zenodo (https://doi.org/10.5281/zenodo.6945203).

Field-specific reporting

Please select the one below that is the best fit for your research. If you are not sure, read the appropriate sections before making your selection.

- [x] Life sciences
- [ ] Behavioural & social sciences
- [ ] Ecological, evolutionary & environmental sciences

For a reference copy of the document with all sections, see nature.com/documents/nr-reporting-summary-flat.pdf

Life sciences study design

All studies must disclose on these points even when the disclosure is negative.

Sample size	Sample size for each library screened was determined by the available size of commercial oligo pool synthesis. Later generations were designed to have larger sample sizes due to performance increase of the algorithm leading to higher confidence for identifying neutral genotypes.
Data exclusions	NGS reads that do not match the designed variants or do not pass quality filtering were excluded as detailed in Methods.
Replication	Two independent replicates were performed using the same condition for each ribozyme variant library screened using NGS. Three independent replicates were performed using PAGE screening for each individually picked variants . Correlation between replicates is shown in the Supplementary Information.
Randomization	Library designed using in silico selection, mutation and recombination were randomized as detailed in Methods.
Blinding	Blinding is not applicable to our study. Model testing were done using held-out data set. The relative activities of each generation of variants were only known after experimental screening.

Reporting for specific materials, systems and methods

We require information from authors about some types of materials, experimental systems and methods used in many studies. Here, indicate whether each material, system or method listed is relevant to your study. If you are not sure if a list item applies to your research, read the appropriate section before selecting a response.

Materials & experimental systems

n/a	Involved in the study	
[x]	[ ]	Antibodies
[x]	[ ]	Eukaryotic cell lines
[x]	[ ]	Palaeontology and archaeology
[x]	[ ]	Animals and other organisms
[x]	[ ]	Human research participants
[x]	[ ]	Clinical data
[x]	[ ]	Dual use research of concern

Methods

n/a	Involved in the study	
[x]	[ ]	ChIP-seq
[x]	[ ]	Flow cytometry
[x]	[ ]	MRI-based neuroimaging
